# Global Distribution of Carbohydrate Utilization Potential in the Prokaryotic Tree of Life

**DOI:** 10.1128/msystems.00829-22

**Published:** 2022-11-22

**Authors:** Rubén López-Mondéjar, Vojtěch Tláskal, Ulisses Nunes da Rocha, Petr Baldrian

**Affiliations:** a Laboratory of Environmental Microbiology, Institute of Microbiology of the Czech Academy of Sciences, Prague, Czech Republic; b CEBAS-CSIC, Department of Soil and Water Conservation, Campus Universitario de Espinardo, Murcia, Spain; c Department of Environmental Microbiology, UFZ-Helmholtz Centre for Environmental Research, Leipzig, Germany; University of California, Davis

**Keywords:** carbohydrate-active enzymes, earth microbiome, natural ecosystems, phylogenetic conservation, habitat specificity

## Abstract

Microorganisms dominate all ecosystems on Earth and play a key role in the turnover of organic matter. By producing enzymes, they degrade complex carbohydrates, facilitating the recycling of nutrients and controlling the carbon cycle. Despite their importance, our knowledge regarding microbial carbohydrate utilization has been limited to genome-sequenced taxa and thus heavily biased to specific groups and environments. Here, we used the Genomes from Earth’s Microbiomes (GEM) catalog to describe the carbohydrate utilization potential in >7000 bacterial and archaeal taxa originating from a range of terrestrial, marine and host-associated habitats. We show that the production of carbohydrate-active enzymes (CAZymes) is phylogenetically conserved and varies significantly among microbial phyla. High numbers of carbohydrate-active enzymes were recorded in phyla known for their versatile use of carbohydrates, such as *Firmicutes*, *Fibrobacterota*, and *Armatimonadota*, but also phyla without cultured representatives whose carbohydrate utilization potential was so far unknown, such as *KSB1*, *Hydrogenedentota*, *Sumerlaeota,* and *UBP3*. Carbohydrate utilization potential reflected the specificity of various habitats: the richest complements of CAZymes were observed in MAGs of plant microbiomes, indicating the structural complexity of plant biopolymers.

**IMPORTANCE** This study expanded our knowledge of the phylogenetic distribution of carbohydrate-active enzymes across prokaryotic tree of life, including new phyla where the carbohydrate-active enzymes composition have not been described until now and demonstrated the potential for carbohydrate utilization of numerous yet uncultured phyla. Profiles of carbohydrate-active enzymes are largely habitat-specific and reflect local carbohydrate availability by selecting taxa with appropriate complements of these enzymes. This information should aid in the prediction of functions in microbiomes of known taxonomic composition and helps to identify key components of habitat-specific carbohydrate pools. In addition, these findings have a high relevance for the understanding of carbohydrate utilization and carbon cycling in the environment, the process that is closely link to the carbon storage potential of Earth habitats and the production of greenhouse gasses.

## INTRODUCTION

Microorganisms are important drivers of decomposition processes occurring in natural habitats, mediating organic carbon (C) turnover and nutrient recycling in all ecosystems on Earth. Through effects on C cycling, they contribute to a range of essential processes, including the control of the performance and health of their plant or animal hosts or the C fluxes at the ecosystem level. Understanding microbial roles in global C models is essential for future predictions of the health of our planet (1–3). Microorganisms degrade complex carbohydrates of various origins found in nature by producing enzymes (4, 5). These microbial enzymes involved in the degradation of C compounds are termed carbohydrate-active enzymes (CAZymes), i.e., enzymes that degrade or modify carbohydrates (http://www.cazy.org/) (6). Most enzymes performing carbohydrate decomposition are glycoside hydrolases (GHs). Furthermore, lytic polysaccharide monooxygenases (LPMOs), oxidases and peroxidases classified as enzymes with auxiliary activities (AAs) in the CAZy database have also been found to play an important role in carbohydrate degradation (7, 8). CAZymes are classified into families and subfamilies based on structural similarity, and this classification reflects to a large extent their substrate specificity (6).

Bacteria and archaea, whose cell numbers on Earth are estimated to be approximately 10^30^, inhabit all types of habitats from soils to the oceans and the sediments of the seabed, living freely or in association with their animal or plant hosts (9, 10). With the advancement of sequencing technologies and bioinformatic resources, first attempts have been made to describe the phylogenetic conservation of microbial enzymes in microbial phylogenies (11, 12) or the occurrence of microbial GHs across environments (13) using limited numbers of genomic and metagenomic data sets that were available at the time of analysis. However, since the potential for carbohydrate utilization of most microbial taxa could not be reliably explored because only a fraction of them have been isolated and cultured (14, 15). This is why our present knowledge of microbial carbohydrate utilization is limited to genome-sequenced taxa and thus heavily biased to specific groups and environments (12). Genome-resolved metagenomics has enabled the reconstruction of microbial genomes from microbial populations. This approach substantially improved our understanding of microbial evolution and enabled the prediction of metabolic capacities of high numbers of relevant microbes across all habitats, including members of novel microbial phyla that lack genome-sequenced isolates (16–20).

Here, we analyzed high-quality metagenome-assembled genomes (MAGs) from the recently compiled Genomes from Earth’s Microbiomes (GEM) catalog, which includes >10,000 metagenomes and >52,000 MAGs from 135 phyla (19). This large-scale genomic inventory provides a critical resource of prokaryotic genomes avoiding taxa isolation biases and linking them with a representative environment on Earth. In the gene models in this catalog, we identified CAZymes involved in the degradation of the main complex carbohydrates in nature, including cellulose, hemicellulose, α-glucans, β-glucans, pectin, chitin, peptidoglycan, and others. We aimed to present a descriptive overview of the abilities of bacterial and archaeal phyla to degrade carbohydrates and to characterize the CAZyme pools of the members of important microbial habitats. While information on CAZyme distribution and phylogenetic conservation should aid in the prediction of functions in microbiomes of known taxonomic composition, the description of habitat-specific enzyme pools helps to identify key components of habitat-specific carbohydrate pools and propose pathways of their transformation (20, 21).

## RESULTS AND DISCUSSION

### Composition and diversity of CAZymes across microbial taxa.

**FIG 1:**
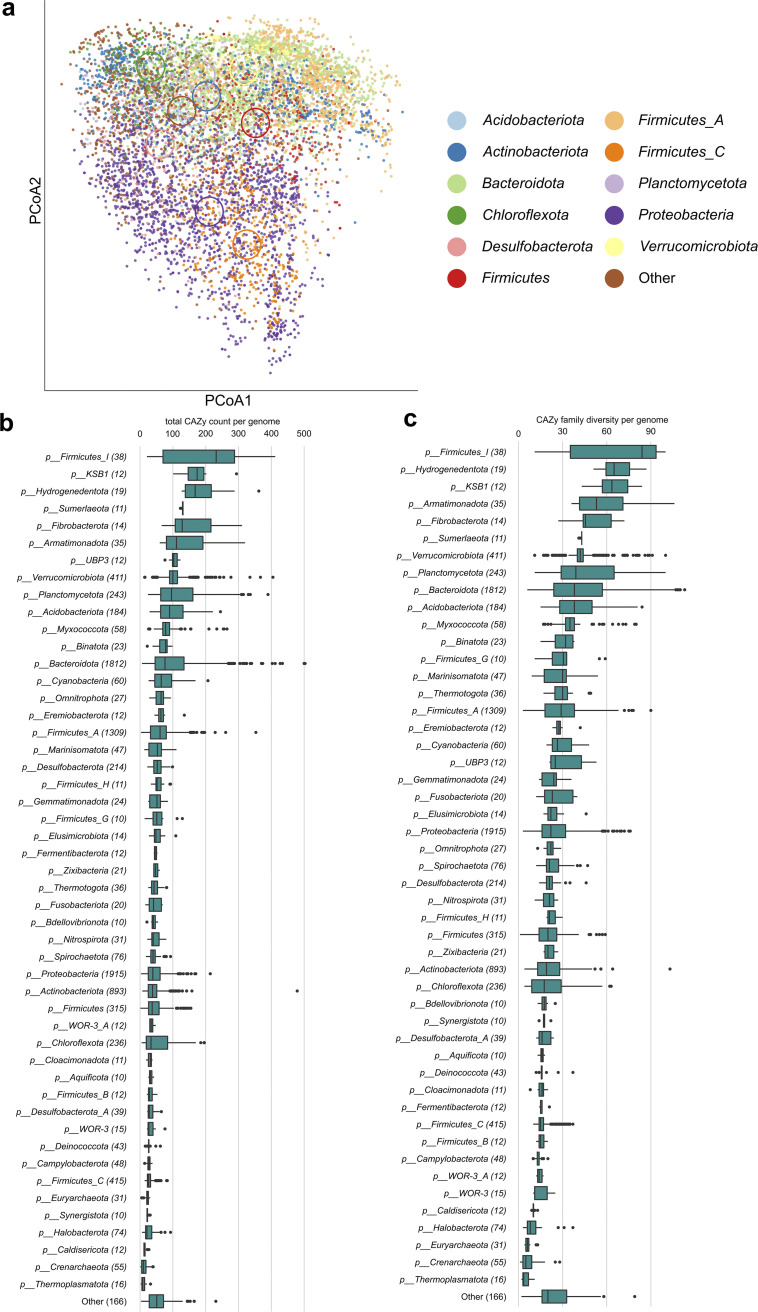
Composition and diversity of carbohydrate-active enzymes of metagenome-assembled genomes across microbial taxa. (a) PCoA based on CAZyme gene pools identified within MAGs. Taxonomic assignments of each MAG (*n* = 9, 143) are color coded. Abundant CAZy types representing >0.1% of all identified CAZymes were considered, and their counts were normalized to construct Euclidean distances. Ellipses represent group centroids. (b–c) Boxplots showing the total CAZy gene count per MAG (CAZy abundance) within phyla and CAZyme functional diversity (number of CAZy families per MAG). The category Other contains phyla with <10 MAGs per phylum (55 phyla with 166 MAGs in total). Numbers in brackets denote MAG count. All CAZymes belonging to the AA, CBM, PL, CE, GH, and GT classes are considered. Boxplots show median values and lower and upper quartiles.

We selected high-quality MAGs (*n* = 9,143) that belonged to 90 bacterial and 14 archaeal phyla. While the potential for carbohydrate utilization was omnipresent along the bacterial and archaeal trees of life, the CAZyme content per genome differed among phyla. The composition of CAZyme pools of *Proteobacteria* and *Firmicutes*_C clustered separately from other major bacterial phyla, although MAGs of both groups still showed a high level of variation in CAZyme composition (Fig. 1a). Among the phyla showing high numbers of CAZymes per genome with a median >100 were some of those well-known for the utilization of a wide range of carbohydrates, such as *Firmicutes*_I, *Fibrobacterota*, and *Armatimonadota* (12, 22), as well as several other phyla uncultured thus far whose carbohydrate utilization potential was so far unknown, such as *KSB1*, *Hydrogenedentota*, *Sumerlaeota,* and *UBP3* (Fig. 1b). Moreover, CAZymes of these phyla also encoded the most diverse complements of CAZyme families per genome, indicating the highest functional diversity (Fig. 1c). When focusing on the most populated phyla, those with the highest CAZy count were *Planctomycetota* (122 ± 5), *Verrucomicrobiota* (106 ± 2), *Acidobacteriota* (99 ± 3), and *Bacteroidota* (97 ± 2). In contrast, proteobacterial taxa contained 46 ± 1 and *Actinobacteriota* 41 ± 1 CAZymes per genome, placing them among average phyla. Another highly populated phylum, *Firmicutes*_C, contained only 28 ± 1 CAZymes per genome, placing it at the low end of the phylum comparison (Fig. 1b). Regarding the content of CAZy families per genome, MAGs of *Planctomycetota* contained genes of 47 ± 1 CAZy families per genome, *Verrucomicrobiota* 44 ± 1, *Bacteroidota* 42 ± 1, and *Acidobacteriota* 41 ± 1. Other phyla, such as *Proteobacteria*, *Actinobacteriota,* and *Firmicutes*_C, contained only 26, 21, and 17 CAZy families per genome, respectively (Fig. 1c).

**FIG 2:**
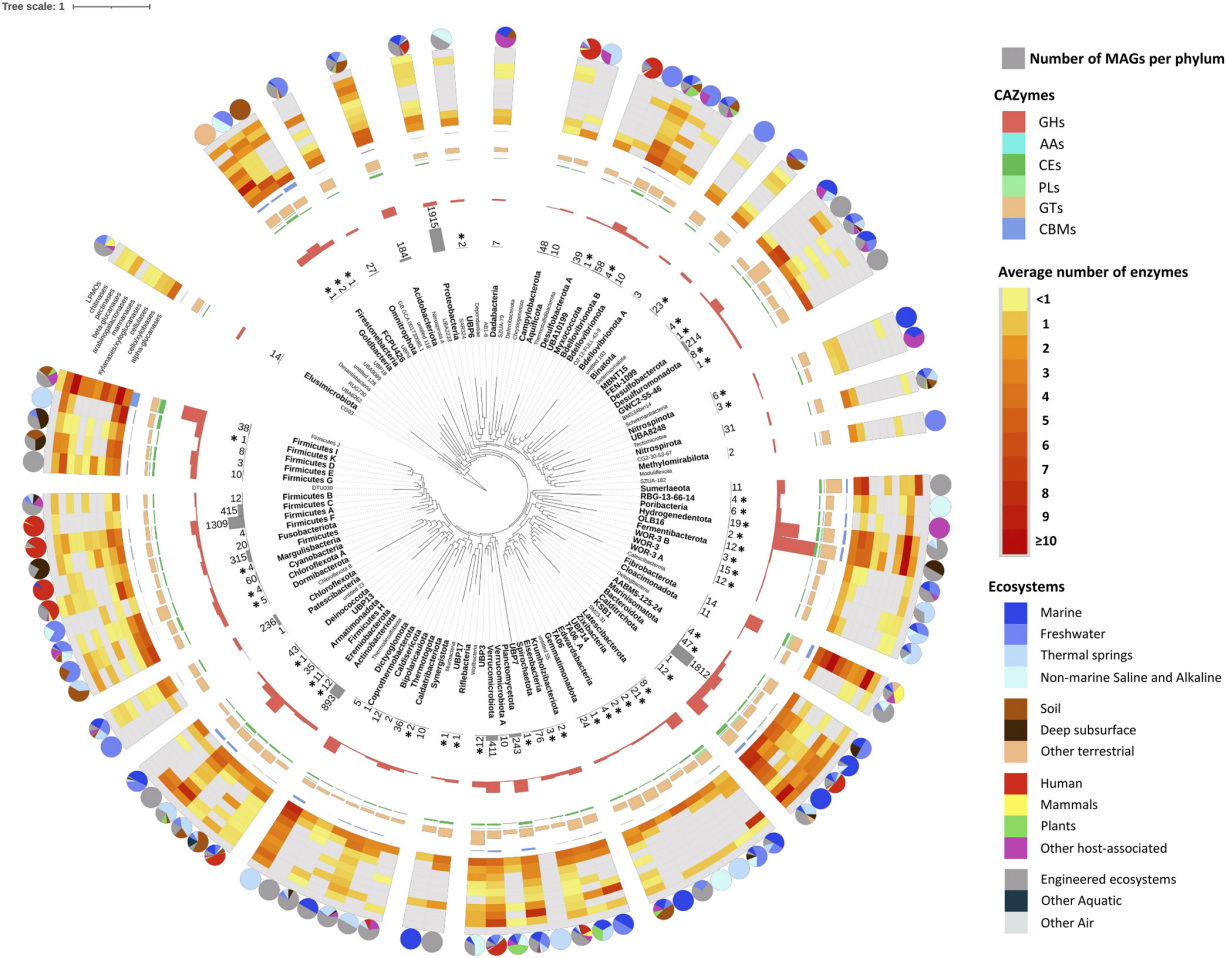
Occurrence and abundance of carbohydrate-active enzymes targeting various carbohydrates in bacterial phyla. Phyla containing at least one MAG are shown in bold. The rings indicate from inside to outside: (i) number of MAGs in the phylum, (ii) content of CAZyme classes, (iii) genomic potential for the degradation of selected carbohydrates, and (iv) habitats from which MAGs originate. Asterisks indicate phyla with previously unrecognized/unexamined potential for carbohydrate degradation. Phylogenetic tree of bacterial phyla was obtained from the Genome Taxonomy Database (GTDB) (https://gtdb.ecogenomic.org/).

Glycoside hydrolases and glycoside transferases (GTs) were found in all 90 bacterial phyla, and carbohydrate esterases (CEs) were found in 88 phyla. Carbohydrate-binding modules (CBMs), polysaccharide lyases (PLs) and AAs were less widespread, occurring in 65, 57, and 17 bacterial phyla, respectively (Fig. 2). The abundance of each class was also different for each taxonomic group. For example, the highest average number of GHs per genome was detected in OLB16 (200 genes), *Hydrogenedentota* (115 genes), *Firmicutes*_I (111 genes), *KSB1* (86 genes), and *Armatimonadota* (76 genes), while the genomes of *Campylobacterota*, *UBP7,* and *Patescibacteria* encoded on average only 3, 2 and 1 GHs, respectively. AAs were generally rare, being more frequent only in the genomes of *Firmicutes*_I, *Myxococcota,* and *Zixibacteria*; in all cases, <1 gene per genome on average. CBMs were most abundant in *Firmicutes*_I, *FCPU426,* and *Fibrobacterota* at 35, 19, and 14 per genome, respectively.

*Bacteroidota*, *Fibrobacterota*, *Acidobacteriota*, *Proteobacteria*, *Verrucomicrobiota*, *Planctomycetota,* and several groups of Firmicutes were identified as the phyla whose members were most versatile in attacking diverse biopolymers of various origins. Species belonging to these taxa are known degraders in diverse natural and engineered ecosystems (12, 22). Interestingly, several less known bacterial phyla can also degrade a high variety of carbohydrates. This is the case for *Goldbacteria*, *Myxococcota*, *Hydrogenedentota*, *OLB16*, *KSB1*, *Dictyoglomota,* and *Firestonebacteria*. A few of these newly described candidate phyla from the rare biosphere were recently reported as carbohydrate degraders (23, 24). Our results now extend this list substantially, as we describe here the ability to degrade carbohydrates in 44 new phyla (Fig. 2), in addition of 46 phyla already included in the CAZY database.

As expected, α-glucanases and cello/xylobiases were highly prevalent, present in 85 and 79 bacterial phyla, respectively. These groups also showed the lower percentage of extracellular enzymes (only 21% α-glucanases and 37% of cello/xylobiases were extracellular) (Fig. S1 in the supplemental material). In contrast, genes involved in the degradation of more recalcitrant or less common biopolymers are less frequent and encode mostly extracellular enzymes (Fig. S1). For example, cellulases were found in 45 phyla (highly abundant in the genomes of *Goldbacteria* and *Fibrobacterota*), chitinases in 63 phyla (most abundant in *Sumerlaeota* and *Verrucomicrobia*), xylanases/xyloglucanases in 31 phyla (abundant in *Firmicutes*_I and *Fibrobacterota*), mannanases in 48 phyla (most abundant in *Goldbacteria* and KSB1), arabinogalactanases in 29 phyla (abundant in *Firmicutes*_I and *Dictyoglomota*), β-glucanases in 58 phyla (most abundant in *Krumholzibacteriota*), and pectinases in 58 phyla (abundant in *Hydrogenedentota* and *OLB16*). LPMOs of the AA10 family were identified only in 7 phyla: *Proteobacteria*, *Bacteroidota*, *Planctomycetota*, *Verrucomicrobiota*, *Actinobacteriota*, *Firmicutes,* and *Firmicutes*_I. Notably, the content of hydrolytic enzymes appears to reflect the lifestyles of some of the less known bacterial phyla, as recently shown for some deep-sea prokaryotes (25).

10.1128/msystems.00829-22.1 FIG S1 Diversity of CAZyme families grouped by activity. Bars indicate the number of genes belonging to that CAZy family. In dark blue, the total number of CAZymes; and in light blue, only the extracellular CAZymes (sequences with signal peptide). Numbers under the figures indicate the percentage of extracellular CAZymes involved in the degradation of that carbohydrate. Download FIG S1, PDF file, 0.2 MB. Copyright © 2022 López-Mondéjar et al. 2022 López-Mondéjar et al. https://creativecommons.org/licenses/by/4.0/ This content is distributed under the terms of the Creative Commons Attribution 4.0 International license.

CAZymes were also found in eight archaeal phyla (*Crenarchaeota*, *Halobacterota*, *Thermoplasmatota*, *Euryarchaeota*, *Hydrothermarchaeota*, *Altiarchaeota*, *Micrarchaeota,* and *UAP2*) (Fig. S2 in the supplemental material). α-Glucanases were found in 6 phyla, cello/xylobiases in three phyla, mannanases and pectinases in two phyla and cellulases, chitinases and β-glucanases in only one phylum, showing relatively limited potential of archaea in carbohydrate degradation (17).

10.1128/msystems.00829-22.2 FIG S2 Occurrence of carbohydrate-active enzymes in archaeal phyla. Phyla containing at least one MAG are shown in bold. The columns and colors indicate from left to right: (i) number of MAGs in the phylum, (ii) content of 7 CAZyme classes, (iii) genomic potential for the degradation of selected carbohydrates, and (iv) habitats from which the MAGs originated. Tree obtained from the Genome Taxonomy Database (GTDB) (https://gtdb.ecogenomic.org/). Download FIG S2, PDF file, 0.2 MB. Copyright © 2022 López-Mondéjar et al. 2022 López-Mondéjar et al. https://creativecommons.org/licenses/by/4.0/ This content is distributed under the terms of the Creative Commons Attribution 4.0 International license.

**FIG 3:**
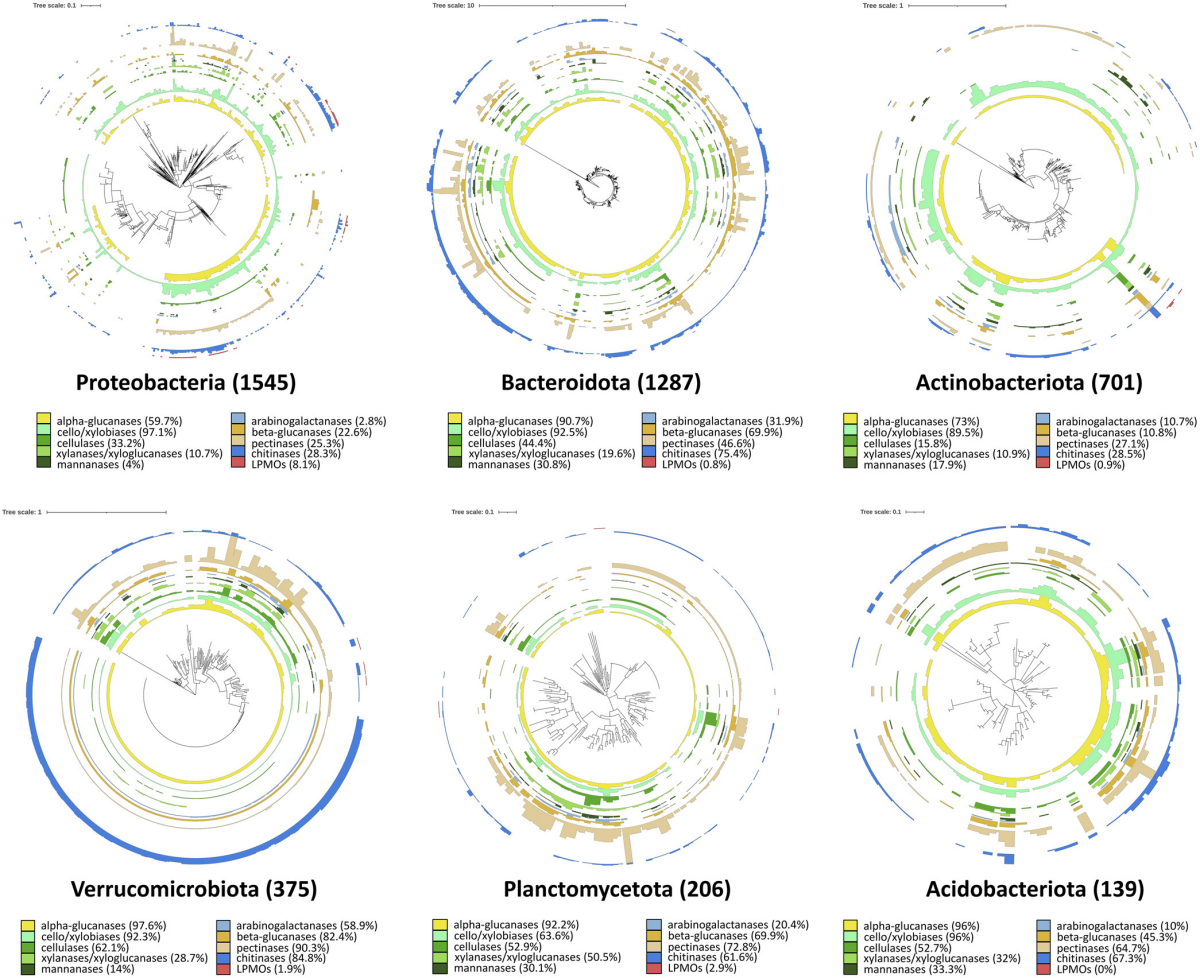
Distribution of CAZymes with various targets across MAGs of six major bacterial phyla. Numbers in brackets represent numbers of MAGs, and percentages indicate the share of MAGs within phyla containing CAZymes with each specific carbohydrate target.

When looking in more detail at individual members of the 19 bacterial phyla with >20 MAGs, a notable variation in carbohydrate utilization potential was observed inside each phylum (Fig. 3, Fig. S3). Chitinases were present in 95% of MAGs of *Marinisomatota* and 90% of *Armatimonadota*, but only in 28% of *Proteobacteria* and *Actinobacteriota*. Cellulases were encoded by the 62% of *Verrucomicrobiota* but only by the 15% of *Actinobacteriota*. In general, cello/xylobiases and α-glucanases were the most common activities encoded by individual taxa, ranging between 60% and 100% of MAGs in all major phyla, confirming previous reports on the ubiquity of these enzymes (12). Among the major bacterial phyla, *Acidobacteriota*, *Planctomycetota,* and *Verrucomicrobiota* appear to be the most capable degraders of biopolymers, with >60% of MAGs possessing genes for the degradation of chitin and pectin and >50% of MAGs possessing cellulases; in *Proteobacteria* and *Actinobacteriota*, corresponding genes were found only in <35% of MAGs (Fig. 3).

10.1128/msystems.00829-22.3 FIG S3 Occurrence of carbohydrate-active enzymes in minor bacterial phyla. Numbers in brackets represent numbers of MAGs, and percentages indicate the share of MAGs within phyla containing CAZymes with each specific carbohydrate target. Download FIG S3, PDF file, 2.1 MB. Copyright © 2022 López-Mondéjar et al. 2022 López-Mondéjar et al. https://creativecommons.org/licenses/by/4.0/ This content is distributed under the terms of the Creative Commons Attribution 4.0 International license.

### Phylogenetic conservation of carbohydrates utilization.

Microbial traits are a product of evolution, and as such, they show various levels of phylogenetic conservation (26, 27). The existence of a phylogenetic signal is an essential factor in microbial ecology since it allows us to assess the probability of certain traits in microbes where trait information from phylogenetically related taxa exists (28, 29). In this sense, the ability to utilize certain classes of carbohydrates (enzymatic activities) can be understood as important microbial traits. We found a significant phylogenetic signal (*P* < 0.001) for the utilization of various carbohydrates in most analyzed phyla (Table S1) with some variation in the mean genetic depth (τ_D_) for each trait (each enzymatic activity) and phylum (Table S2, Fig. S4). The percentage of divergence in the 16S rRNA gene for significantly conserved traits varied for the different activities inside the same phylum. For example, values ranged between 3 to 10% in *Verrucomicrobiota* and 2 to 8% in *Actinobacteriota* and between 1 to 3% in *Proteobacteria* and 0.1 to 0.5% in *Firmicutes*_A. In general, the highest significance of the trait-phylogeny association was found for β-glucanases and cellulases (significant for 73% and 63% of the tested phyla, respectively), and the lowest significance was obtained for α-glucanases and cello/xylobiases (25% and 32%, respectively). Previous observations showed phylogenetic clustering of a few selected traits at a relatively fine scale (11, 30). Our results now vastly extend this information to a wide range of microbial phyla and multiple carbohydrate utilization traits, showing the level of phylogenetic predictability.

10.1128/msystems.00829-22.4 FIG S4 Phylogenetic conservation of carbohydrate utilization traits in microorganisms. Heatmap showing the mean genetic depth (τD) of the consensus clades sharing the ability to degrade different carbohydrates in the selected phyla (phyla including more than 20 retrieved MAGs are shown). Only significant values (*P* < 0.05) of the trait-phylogeny association are shown. Download FIG S4, PDF file, 0.2 MB. Copyright © 2022 López-Mondéjar et al. 2022 López-Mondéjar et al. https://creativecommons.org/licenses/by/4.0/ This content is distributed under the terms of the Creative Commons Attribution 4.0 International license.

10.1128/msystems.00829-22.6 TABLE S1 Significance of the phylogenetic distance of the genes involved in the utilization of different carbohydrates in several bacterial phyla. Abouheif's Cmean was utilized to assess the phylogenetic signal of quantitative variables (*P* values <0.05 are considered statistically significant). Activities statistically significant in each phylum are marked in bold. Download Table S1, DOCX file, 0.03 MB. Copyright © 2022 López-Mondéjar et al. 2022 López-Mondéjar et al. https://creativecommons.org/licenses/by/4.0/ This content is distributed under the terms of the Creative Commons Attribution 4.0 International license.

10.1128/msystems.00829-22.7 TABLE S2 Extent of the phylogenetic conservation of carbohydrate utilization in bacterial phyla based on the consenTRAIT algorithm. The mean genetic depth (τD) of the consensus clades sharing the ability to degrade certain carbohydrate class, the significance (Npermutations = 1,000, *P*-value) of the trait-phylogeny association and the percentage of divergence in the 16S rRNA gene for significantly conserved traits. Trait and values in bold indicate significant trait conservation (*P* < 0.05). Download Table S2, DOCX file, 0.04 MB. Copyright © 2022 López-Mondéjar et al. 2022 López-Mondéjar et al. https://creativecommons.org/licenses/by/4.0/ This content is distributed under the terms of the Creative Commons Attribution 4.0 International license.

### Composition and diversity of CAZymes across habitats.

**FIG 4:**
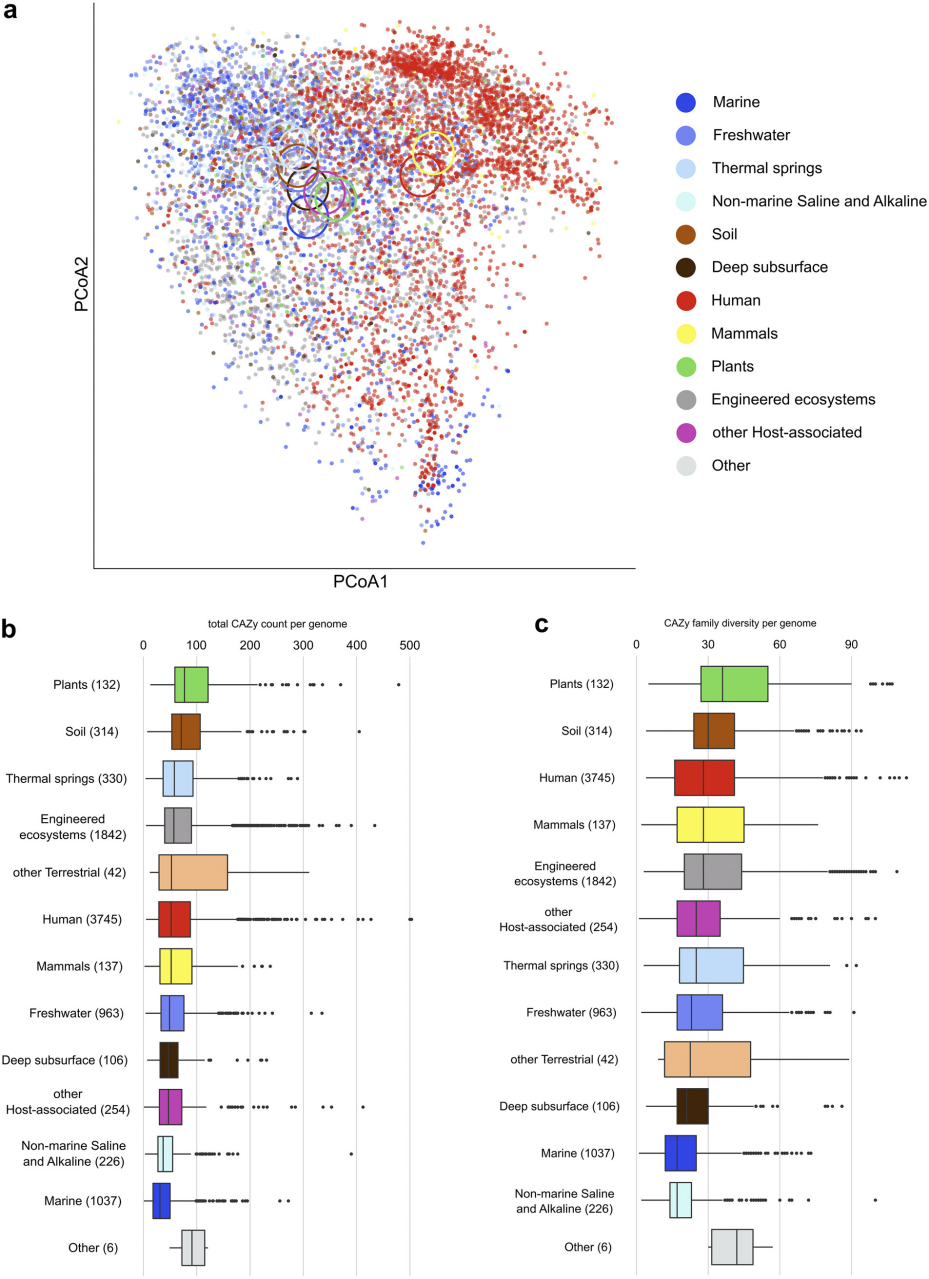
Composition and diversity of carbohydrate-active enzymes of metagenome-assembled genomes across habitats. (a) PCoA based on CAZymes identified within MAGs. Habitat occurrence of each MAG (*n* = 9,143) is color coded. Abundant CAZy types representing >0.1% of all identified CAZymes were considered, and their counts were normalized to construct Euclidean distances. Ellipses represent group centroids. (b–c) Boxplots showing total CAZy gene count per MAG (CAZy abundance) within habitat and CAZyme functional diversity (number of CAZy families per MAG). Numbers in brackets denote MAG count. All CAZymes belonging to the AA, CBM, PL, CE, GH, and GT classes are considered. Boxplots show median values and lower and upper quartiles.

Habitat specificity in CAZyme pool composition was previously observed when comparing environmental metagenomes (13, 31). Here, we show that a certain level of this habitat specificity of CAZyme pools indeed exists: CAZyme pools of MAGs from human and mammalian microbiomes clustered together and separately from those from other habitats. CAZyme pools of MAGs from terrestrial, aquatic and engineered habitats and MAGs from plant microbiomes, however, primarily overlapped (Fig. 4a), confirming that the CAZy content in microbes is more affected by phylogeny than by habitat. The differences in CAZyme profiles among habitats are thus driven by differences in the phylogenetic composition of microbiomes among habitats rather than by the selection of strains with specific traits. The richest complements of CAZymes were observed in MAGs of plant microbiomes, with 106 CAZymes belonging to 43 families on average followed by soils (88 CAZymes in 36 families); the MAGs from marine habitats were less rich in CAZymes, with 39 CAZyme genes in 20 families per MAG on average (Fig. 4b and c). These differences most likely indicate the diversity and structural complexity of carbohydrates in each habitat, with the lignocellulose of vascular plants representing the carbohydrate pool of the highest complexity (32).

**FIG 5:**
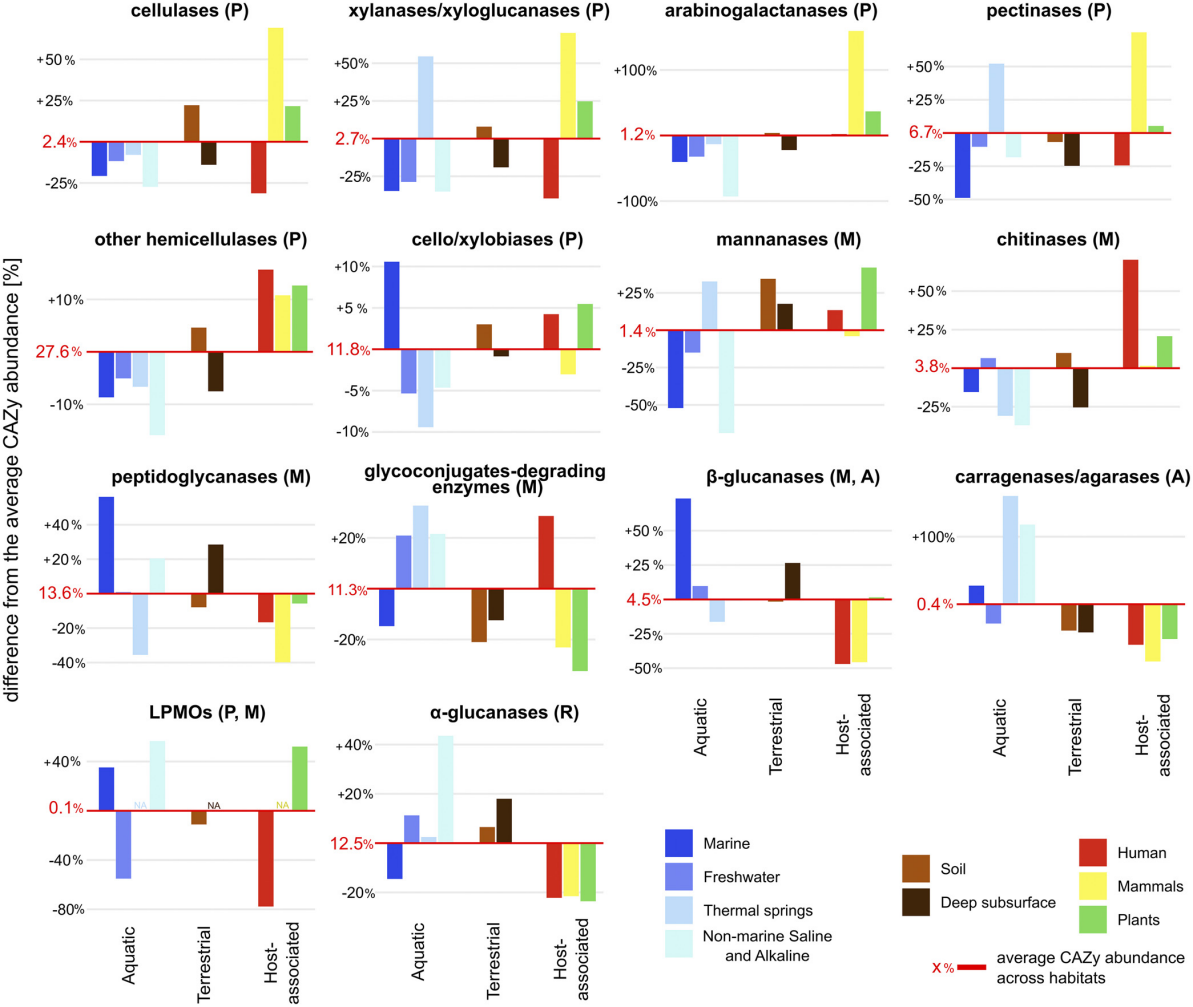
Difference in the relative abundance of CAZymes targeting various carbohydrates across habitats. Mean relative abundances of CAZymes across all habitats are indicated by a red line and a percentage. They represent a share of CAZyme genes targeting certain carbohydrates of all CAZyme genes where targets can be reliably assigned. Differences in the relative abundance across habitats reflect relative enrichment compared with the mean relative abundance. Capital letters close to the activity indicate the main origin of compounds attacked by each activity: P, plant origin; M, microbial origin; A, algal origin; and R, reserve compounds.

Among those CAZymes where substrate specificity can be reliably assigned to target carbohydrates, genes targeting carbohydrates of plant origin (cellulose, hemicelluloses and pectin) were most abundant. In addition, the share of CAZymes targeting microbial biomass and reserve compounds was also high (Fig. 5). Notably, genes targeting the globally most abundant biopolymers, cellulose and chitin were targeted by only 2.4% and 3.8% of such CAZymes, respectively. The composition of CAZyme pools differed significantly among habitats (*P* < 0.001) and reflected the carbohydrate sources in various habitats. The plant-associated microbiomes, rumen microbiomes, and soils were enriched in genes targeting cellulose, xylan, pectin, and other plant-derived compounds. These habitats appear to be potential gold mines for strains and genes of interest in lignocellulosic biomass conversion processes (22, 33, 34). The gut microbiomes of humans showed a lower proportion of plant targets reflecting different diets (Fig. 5). Not surprisingly, marine microbiomes rich in algal biomass showed a higher share of carragenases, agarases, and β-glucanases, supporting the assumption that β-glucan laminarin and other algal compounds are major molecules in the marine C cycle (35). α-Glucanases showed abundance in the deep subsurface, soil and nonmarine saline and alkaline habitats while being less abundant in host-associated habitats and seem to reflect the higher temporal fluctuation of C supply where energy reserves are important for survival (36). Their increased share in freshwater indicates the importance of this habitat in the mineralization of organic C (37, 38). Habitat specificity was also observed at the level of the gene abundances of dominant CAZyme families: while plant and mammalian microbiomes were enriched in selected cellulases and xylanases, soil and freshwater microbiomes showed enrichment of α-glucanases, indicating the importance of utilization of reserve compounds (Fig. S5).

10.1128/msystems.00829-22.5 FIG S5 Relative abundance of genes from the top 30 CAZyme families in selected habitats. We considered all CAZymes with assigned functions. Dots display average relative abundance across all habitats. Download FIG S5, PDF file, 0.2 MB. Copyright © 2022 López-Mondéjar et al. 2022 López-Mondéjar et al. https://creativecommons.org/licenses/by/4.0/ This content is distributed under the terms of the Creative Commons Attribution 4.0 International license.

### Conclusions.

Despite the known limitations of genomes reconstructed from shotgun metagenomes (19), the use of high-quality MAGs allows an in-depth analysis of the prokaryotic tree of life, overcoming the taxonomic limitations of isolation-biased genomes available in other databases and offering also a direct link with a specific environment. Our results provide a comprehensive catalog of carbohydrate-active enzymes across microbial phyla, including new phyla where the CAZyme composition has not been described until now: 49% of the phyla with CAZYmes reported in this study are not yet listed in the CAZY database. We also demonstrate the potential for carbohydrate utilization of numerous yet uncultured phyla, making up 57% of the phyla reported in this study. We show that the composition of the CAZyme pool is phylogenetically conserved across bacterial phyla. Profiles of CAZymes are largely habitat-specific and reflect local carbohydrate availability by selecting taxa with appropriate complements of CAZymes.

## MATERIALS AND METHODS

### CAZyme annotation.

MAGs from the Genomes from Earth’s Microbiomes (GEM) catalog classified as high-quality (*n* = 9,143) were filtered from the published set of total MAGs (*n* = 52,515) (19). Their study defined MAGs as high-quality based on the presence of a near-full complement of rRNAs, tRNAs and single-copy protein-coding genes. CAZymes were predicted using the amino acid sequences of predicted genes of high-quality MAGs that served as the input into the *run_dbcan.py* (v2.0.11) program (39) and were compared with the dbCAN database V8 (40) using HMMER 3.3 (41). CAZymes predicted with a confidence threshold E value ≤ 1E-20 were considered correctly annotated and used further; genes annotated to CAZymes, but without known previous occurrence in bacteria or archaea were omitted. Prediction of function and substrate specificity of CAZyme families or subfamilies was performed based on a review of activities assigned to CAZymes with known structures (characterized enzymes) in the CAZy database (http://www.cazy.org) (6) and manually curated. The CAZyme families (or subfamilies where present) were grouped based on their main characterized enzymatic activities into 14 target carbohydrate classes or general activities according to their main target: (i) cellulases (acting on cellulose), (ii) xylanases/xyloglucanases (acting on main chain of xylans and xyloglucans from plant biomass), (iii) β-glucosidases/β-xylosidases (acting on oligomers from plant origin such as cellobiose and xylobiose), (iv) chitinases/chitosanases (acting on chitin, chitobiose and chitosan), (v) α-glucanases (acting on glucans linked by α-glycosidic bonds), (vi) β-glucanases (acting in glucans linked by different β-linkages), (vii) mannanases (acting on polymers and dimers of mannan), (viii) arabinogalactanases (acting on the links between galactans and arabinans in arabinogalactans from plant origin), (ix) other hemicellulases (acting on side groups such as arabinoses, galactoses or acetyl groups present in hemicelluloses), (x) pectinases (acting on pectin from plant origin), (xi) carragenases/agarases (acting in several polymers from algal origin), (xii) peptidoglycanases (degrading peptidoglycan), (xiii) glycoconjugate-degrading enzymes (CAZymes acting on glycoproteins, glycolipids or proteoglycans), and (xiv) lytic polysaccharide monoxygenases (LPMOs). The CAZyme families/subfamilies belonging to each of these classes or general activities are shown in detail in Table S3.

10.1128/msystems.00829-22.8 TABLE S3 The CAZyme families/subfamilies were grouped according to the main enzymatic activities characterized in the family/subfamily into 14 different classes or general activities. Download Table S3, DOCX file, 0.04 MB. Copyright © 2022 López-Mondéjar et al. 2022 López-Mondéjar et al. https://creativecommons.org/licenses/by/4.0/ This content is distributed under the terms of the Creative Commons Attribution 4.0 International license.

### Phylogenetic tree construction.

Nucleotide sequences of those individual MAGs that contained annotated 16S rRNA gene sequences served as the input to RNAMMER 1.2 (42). In total, 7,162 of the high-quality MAGs contained one copy of 16S rRNA; the rest of the high-quality MAGs with multiple variants of 16S rRNA (*n* = 207) and with missing 16S rRNA (*n* = 1,774) were omitted from further phylogenetic analyses. Sequences of MAGs belonging to the same phylum were aligned with MAFFT (43). Phylogenetic trees were constructed via the maximum likelihood method (ML) using a Kimura 2-parameter Model (K2) and a discrete gamma distribution with invariant sites (G+I) (bootstrap confidence levels determined by 500 bootstrap replications) with the software package MEGA7 (44).

### Statistical analyses.

Source metagenomes and taxonomy of MAGs were retrieved from published catalog (19) and used to calculate the distribution of CAZymes across habitats and taxonomic groups. The influence of MAG taxonomy and habitat occurrence on CAZyme counts was tested using permutational multivariate analysis of variance in *vegan* (v2.5 to 6) function adonis (45), and CAZy families representing >0.1% of all identified CAZymes were considered for principal coordinate analysis. Their counts in individual MAGs were normalized using function decostand and we used the “normalize” option, which made the sum of squares for each MAG equal to one, to construct a dissimilarity matrix with Euclidean distances that served as the input into the cmdscale function in the package stats (v4.0.2) (46). Ellipses representing group centroids were drawn for each phylum or habitat. The differences between the main activities across the habitats were also tested by permutational multivariate analysis of variance using pairwise.adonis(), a wrapper function for multilevel pairwise comparison using adonis in vegan. Signal peptides in the annotated CAZymes were detected using SignalP 6.0 (47). The phylosignal package (v1.3) (48) was used to calculate phylogenetic conservatism within bacterial phyla using Newick-formatted 16S rRNA phylogenetic trees and lists of related CAZymes. We used the “phyloSignal” function for estimating Abouheif's Cmean (49) statistics.

We applied a consenTRAIT analysis (26) using the package *castor* (v1.6.4) (50) to calculate the mean phylogenetic depth (τ_D_) at which traits (i.e., the ability to decompose certain class of carbohydrates) are conserved across clades in the phylogenetic trees of the bacterial phyla. The consenTRAIT algorithm identifies phylogenetic clades that are positive for the trait and calculates the average depth of those clades from a phylogenetic tree. The average phylogenetic depth of the positive clades was compared with the same values calculated after randomizing the responses among the tips 1,000 times to determine whether the trait and phylogeny were significantly nonrandomly associated. The probability of phylogenetic conservation (nonrandomness) of the trait distribution was calculated as the fraction of simulated τ_D_ values that were greater than or equal to the observed τ_D_. To obtain a measure of the sequence identity of organisms with a positive clade, τ_D_ was multiplied by two and then subtracted from 1. These values are comparable to a cutoff for defining operational taxonomic units (11).

### Figure generation.

Manuscript figures were generated using custom R scripts (46), Rstudio (51), tidyverse (52), Inkscape (https://inkscape.org/), and iTOL (53).

### Data availability.

The MAG data used in this study were published in the previous paper (19). Bulk download for their 52,515 MAGs is available at https://genome.jgi.doe.gov/GEMs and https://portal.nersc.gov/GEM. Annotation of CAZymes in the high-quality MAGs is available at Figshare, https://doi.org/10.6084/m9.figshare.16435581. The code for reproducing gene calling and the annotation of CAZymes is provided at https://github.com/TlaskalV/Global-CAZymes.
