# Supplementary figures and images for: Global Distribution of Carbohydrate Utilization Potential in the Prokaryotic Tree of Life
Source: mSystems. 2022 Nov 22;7(6):e00829-22. doi: 10.1128/msystems.00829-22 (PMC9765126; doi:10.1128/msystems.00829-22)

Supplemental Figure S1

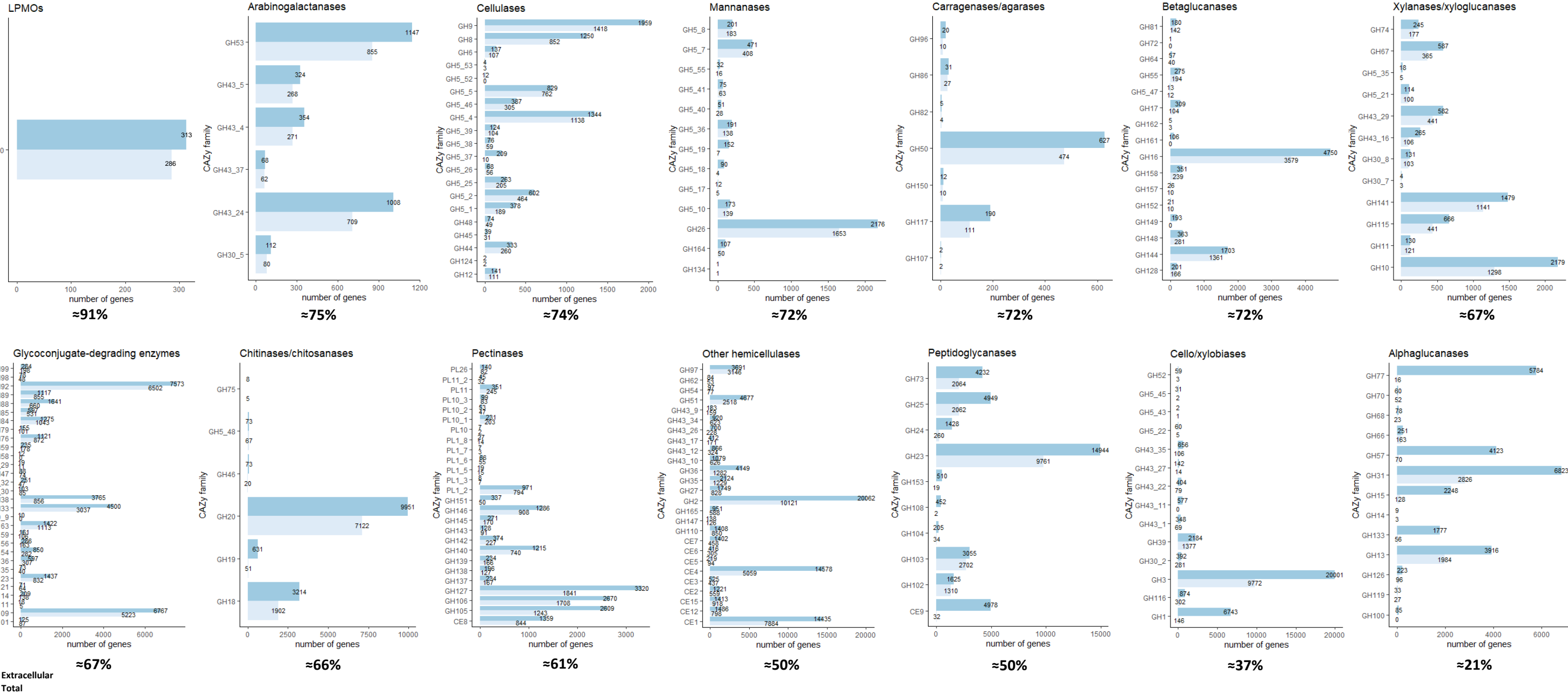

Supplement: FIG S1 [file msystems.00829-22-s0001.pdf]

## Supplemental Figure S4

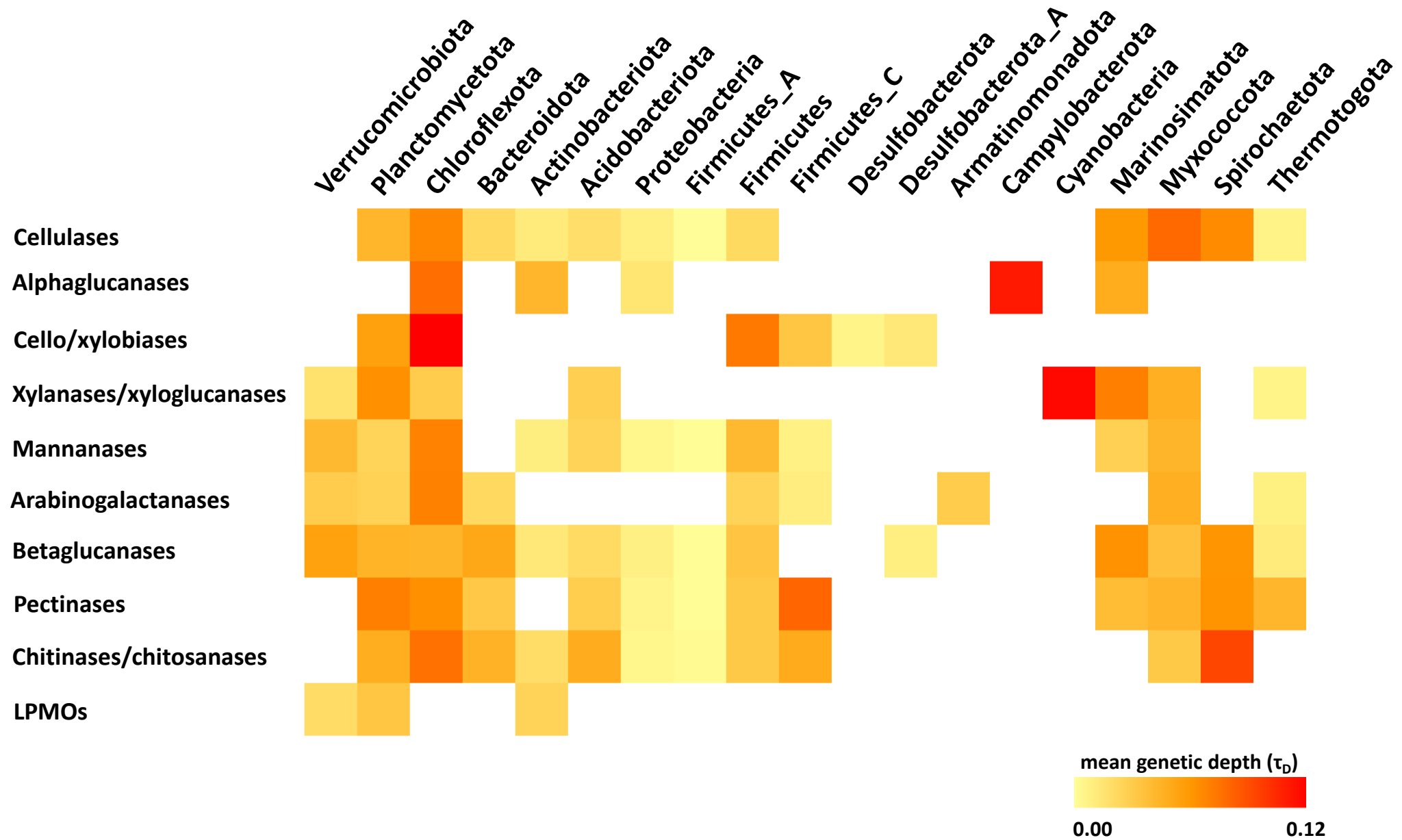

Supplement: FIG S4 [file msystems.00829-22-s0004.pdf]

Supplemental Figure S5

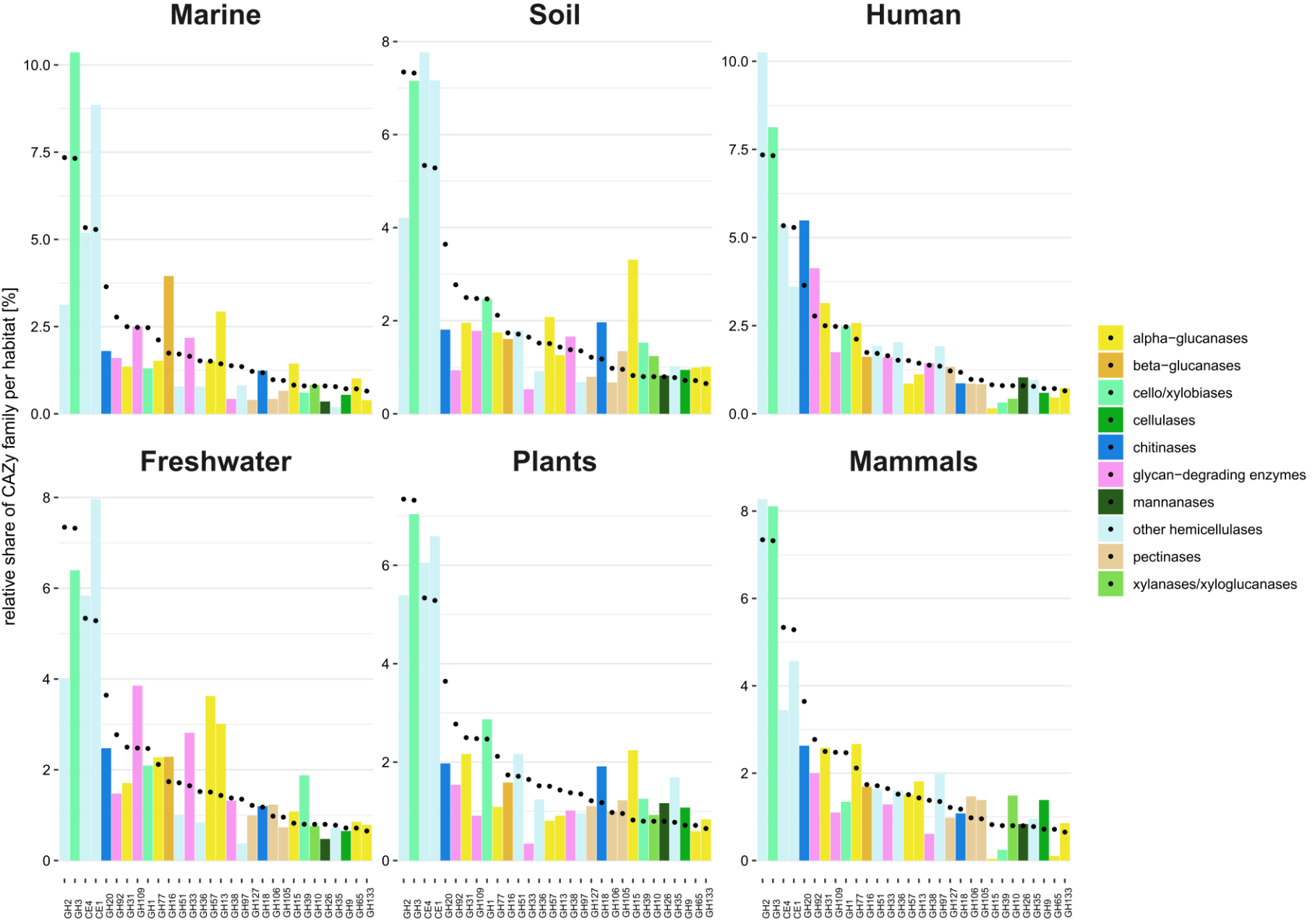

Supplement: FIG S5 [file msystems.00829-22-s0005.pdf]
