# Supplementary material for: Global Distribution of Carbohydrate Utilization Potential in the Prokaryotic Tree of Life
Source: mSystems. 2022 Nov 22;7(6):e00829-22. doi: 10.1128/msystems.00829-22 (PMC9765126; doi:10.1128/msystems.00829-22)

Supplemental Figure S3

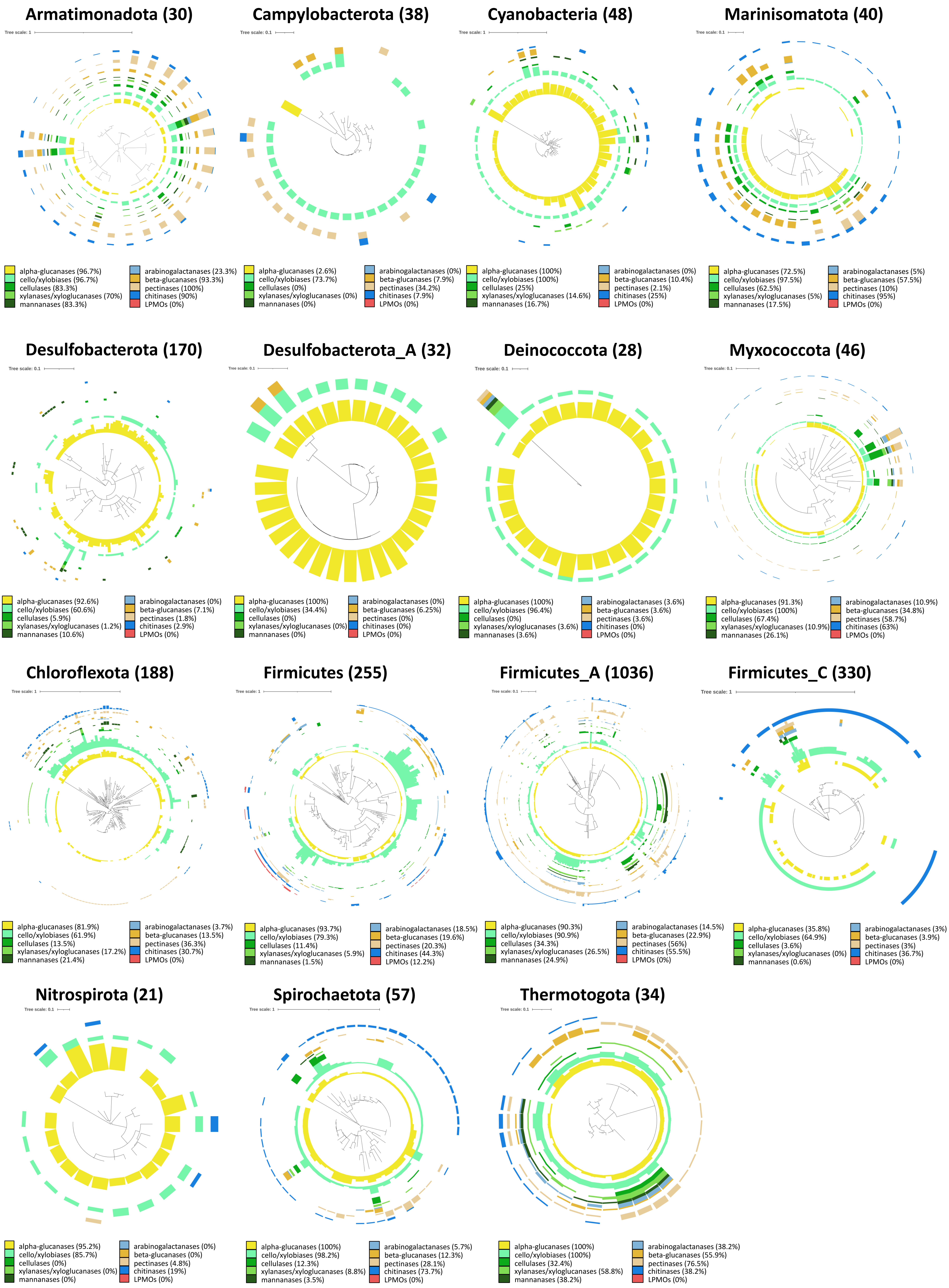

Supplement: FIG S3 [file msystems.00829-22-s0003.pdf]
