# Supplementary material for: Global Distribution of Carbohydrate Utilization Potential in the Prokaryotic Tree of Life
Source: mSystems. 2022 Nov 22;7(6):e00829-22. doi: 10.1128/msystems.00829-22 (PMC9765126; doi:10.1128/msystems.00829-22)
Supplement: TABLE S1 [file msystems.00829-22-s0006.docx]

Supplementary Table S1. Significance of the phylogenetic distance of the genes involved in the utilization of different carbohydrates in several bacterial phyla. Abouheif's Cmean was utilized to assess the phylogenetic signal of quantitative variables (P-values <0.05 are considered statistically significant). Activities statistically significant in each phylum are marked in bold.

| Phylum | Enzymes | C_mean_ | P-value |
| --- | --- | --- | --- |
| Verrucomicrobiota | **cellulases** | 0.4392919 | 0.001 |
|  | **alphaglucanases** | 0.4585063 | 0.001 |
|  | **cello/xylobiases** | 0.4834084 | 0.001 |
|  | **chitinases** | 0.9499268 | 0.001 |
|  | **xylanases/xyloglucanases** | 0.4752826 | 0.001 |
|  | **pectinases** | 0.9499268 | 0.001 |
|  | **LPMOs** | 0.5262702 | 0.001 |
| Planctomycetota | **cellulases** | 0.5952123 | 0.001 |
|  | **alphaglucanases** | 0.6296811 | 0.001 |
|  | **cello/xylobiases** | 0.5952123 | 0.001 |
|  | **chitinases** | 0.3048851 | 0.001 |
|  | **xylanases/xyloglucanases** | 0.7788384 | 0.001 |
|  | **pectinases** | 0.5771256 | 0.001 |
|  | **LPMOs** | 0.3998033 | 0.001 |
| Chloroflexota | **cellulases** | 0.3262649 | 0.001 |
|  | **alphaglucanases** | 0.6006642 | 0.001 |
|  | **cello/xylobiases** | 0.7930266 | 0.001 |
|  | **chitinases** | 0.6150652 | 0.001 |
|  | **xylanases/xyloglucanases** | 0.2260442 | 0.002 |
|  | **pectinases** | 0.4008385 | 0.001 |
|  | LPMOs | - | - |
| Bacteroidota | **cellulases** | 0.5673848 | 0.001 |
|  | **alphaglucanases** | 0.6882671 | 0.001 |
|  | **cello/xylobiases** | 0.7096114 | 0.001 |
|  | **chitinases** | 0.810141 | 0.001 |
|  | **xylanases/xyloglucanases** | 0.556013 | 0.001 |
|  | **pectinases** | 0.5703934 | 0.001 |
|  | **LPMOs** | 0.6651945 | 0.001 |
| Actinobacteriota | **cellulases** | 0.3035073 | 0.001 |
|  | **alphaglucanases** | 0.7277892 | 0.001 |
|  | **cello/xylobiases** | 0.7285253 | 0.001 |
|  | **chitinases** | 0.4927954 | 0.001 |
|  | **xylanases/xyloglucanases** | 0.4579571 | 0.001 |
|  | **pectinases** | 0.5395931 | 0.001 |
|  | **LPMOs** | 0.5004626 | 0.001 |
| Acidobacteriota | **cellulases** | 0.5205703 | 0.001 |
|  | **alphaglucanases** | 0.6379647 | 0.001 |
|  | **cello/xylobiases** | 0.5989924 | 0.001 |
|  | **chitinases** | 0.4964685 | 0.001 |
|  | **xylanases/xyloglucanases** | 0.5917379 | 0.001 |
|  | **pectinases** | 0.4565847 | 0.001 |
| Phylum | Enzymes | C_mean_ | P-value |
| Acidobacteriota | LPMOs | - | - |
| Proteobacteria | **cellulases** | 0.5717478 | 0.001 |
|  | **alphaglucanases** | 0.8122067 | 0.001 |
|  | **cello/xylobiases** | 0.777551 | 0.001 |
|  | **chitinases** | 0.6841675 | 0.001 |
|  | **xylanases/xyloglucanases** | 0.5181393 | 0.001 |
|  | **pectinases** | 0.5128865 | 0.001 |
|  | **LPMOs** | 0.6906509 | 0.001 |
| Firmicutes_A | **cellulases** | 0.7740129 | 0.001 |
|  | **alphaglucanases** | 0.719741 | 0.001 |
|  | **cello/xylobiases** | 0.8158747 | 0.001 |
|  | **chitinases** | 0.6736458 | 0.001 |
|  | **xylanases/xyloglucanases** | 0.7858945 | 0.001 |
|  | **pectinases** | 0.5896569 | 0.001 |
|  | LPMOs | - | - |
| Firmicutes | **cellulases** | 0.06518463 | 0.045 |
|  | **alphaglucanases** | 0.5261866 | 0.001 |
|  | **cello/xylobiases** | 0.8424325 | 0.001 |
|  | **chitinases** | 0.6452947 | 0.001 |
|  | **xylanases/xyloglucanases** | 0.2950863 | 0.001 |
|  | **pectinases** | 0.640768 | 0.001 |
|  | **LPMOs** | 0.7350063 | 0.001 |
| Firmicutes_C | **cellulases** | 0.6090167 | 0.001 |
|  | **alphaglucanases** | 0.5766505 | 0.001 |
|  | **cello/xylobiases** | 0.7278312 | 0.001 |
|  | **chitinases** | 0.9170037 | 0.001 |
|  | xylanases/xyloglucanases | - | - |
|  | **pectinases** | 0.842631 | 0.001 |
|  | LPMOs | - | - |
| Desulfobacterota | **cellulases** | 0.1636391 | 0.016 |
|  | **alphaglucanases** | 0.5179253 | 0.001 |
|  | **cello/xylobiases** | 0.3861175 | 0.001 |
|  | chitinases | -0.02764696 | 0.582 |
|  | xylanases/xyloglucanases | -0.01070392 | 0.759 |
|  | pectinases | -0.01262723 | 0.186 |
|  | LPMOs | - | - |
| Desulfobacterota_A | cellulases | - | - |
|  | **alphaglucanases** | 0.8859026 | 0.001 |
|  | **cello/xylobiases** | 0.7854481 | 0.001 |
|  | chitinases | - | - |
|  | xylanases/xyloglucanases | - | - |
|  | pectinases | - | - |
|  | LPMOs | - | - |
| Armatinomonadota | **cellulases** | 0.1860595 | 0.047 |
|  | **alphaglucanases** | 0.4943427 | 0.001 |
|  | **cello/xylobiases** | 0.2646152 | 0.025 |
| Phylum | Enzymes | C_mean_ | P-value |
| Armatinomonadota | **chitinases** | 0.5358766 | 0.001 |
|  | xylanases/xyloglucanases | -0.07303066 | 0.585 |
|  | **pectinases** | 0.4834364 | 0.001 |
|  | LPMOs | - | - |
| Campylobacterota | cellulases | - | - |
|  | alphaglucanases | -0.0244033 | 0.027 |
|  | **cello/xylobiases** | 0.4552443 | 0.001 |
|  | chitinases | -0.07293718 | 0.604 |
|  | xylanases/xyloglucanases | - | - |
|  | **pectinases** | 0.5327944 | 0.001 |
|  | LPMOs | - | - |
| Cyanobacteria | cellulases | 0.04956747 | 0.204 |
|  | **alphaglucanases** | 0.3707171 | 0.001 |
|  | **cello/xylobiases** | 0.3563333 | 0.001 |
|  | **chitinases** | 0.2995019 | 0.002 |
|  | xylanases/xyloglucanases | 0.1309409 | 0.072 |
|  | pectinases | -0.02130485 | 0.327 |
|  | LPMOs | - | - |
| Deinococcota | cellulases | - | - |
|  | alphaglucanases | 0.2860624 | 0.005 |
|  | cello/xylobiases | -0.01913613 | 0.171 |
|  | chitinases | - | - |
|  | **xylanases/xyloglucanases** | -0.03246827 | 0.042 |
|  | **pectinases** | -0.03246827 | 0.039 |
|  | LPMOs | - | - |
| Marinisomatota | **cellulases** | 0.4816683 | 0.001 |
|  | **alphaglucanases** | 0.7722841 | 0.001 |
|  | **cello/xylobiases** | 0.3687679 | 0.001 |
|  | **chitinases** | 0.6368661 | 0.001 |
|  | **xylanases/xyloglucanases** | 0.4846383 | 0.004 |
|  | **pectinases** | 0.482177 | 0.003 |
|  | LPMOs | - | - |
| Myxococcota | **cellulases** | 0.4604105 | 0.004 |
|  | **alphaglucanases** | 0.5694153 | 0.001 |
|  | **cello/xylobiases** | 0.2678183 | 0.015 |
|  | **chitinases** | 0.2713104 | 0.001 |
|  | xylanases/xyloglucanases | 0.06108171 | 0.083 |
|  | **pectinases** | 0.4105637 | 0.002 |
|  | LPMOs | - | - |
| Nitrospirota | cellulases | - | - |
|  | alphaglucanases | -0.1725373 | 0.842 |
|  | cello/xylobiases | 0.01494674 | 0.295 |
|  | chitinases | -0.06581125 | 0.426 |
|  | xylanases/xyloglucanases | - | - |
|  | pectinases | -0.05046852 | 0.387 |
|  | LPMOs | - | - |
| Phylum | Enzymes | C_mean_ | P-value |
| Spirochaetota | **cellulases** | 0.2138242 | 0.028 |
|  | **alphaglucanases** | 0.5813082 | 0.001 |
|  | **cello/xylobiases** | 0.1921723 | 0.024 |
|  | **chitinases** | 0.4728607 | 0.001 |
|  | xylanases/xyloglucanases | -0.03851098 | 0.436 |
|  | **pectinases** | 0.5325815 | 0.001 |
|  | LPMOs | - | - |
| Thermotogota | **cellulases** | 0.7182233 | 0.001 |
|  | **alphaglucanases** | 0.6609254 | 0.001 |
|  | **cello/xylobiases** | 0.4400532 | 0.001 |
|  | **chitinases** | 0.6595647 | 0.001 |
|  | **xylanases/xyloglucanases** | 0.6744308 | 0.001 |
|  | **pectinases** | 0.6736998 | 0.001 |
|  | LPMOs | - | - |
