# Supplementary material for: Global Distribution of Carbohydrate Utilization Potential in the Prokaryotic Tree of Life
Source: mSystems. 2022 Nov 22;7(6):e00829-22. doi: 10.1128/msystems.00829-22 (PMC9765126; doi:10.1128/msystems.00829-22)
Supplement: TABLE S2 [file msystems.00829-22-s0007.docx]

Supplementary Table S2. Extent of the phylogenetic conservation of carbohydrate utilization in bacterial phyla based on the consenTRAIT algorithm. The mean genetic depth (τD) of the consensus clades sharing the ability to degrade certain carbohydrate class, the significance (Npermutations=1,000, P-value) of the trait-phylogeny association and the percentage of divergence in the 16S rRNA gene for significantly conserved traits. Trait and values in bold indicate significant trait conservation (P<0.05).

| **Phyla** | **Trait** | **Mean genetic depth (τ_D_)** | ***P*-value** | **Percentage of divergence in the 16S rRNA gene** |
| --- | --- | --- | --- | --- |
| Verrucomicrobiota | cellulases | 0.00943283 | 0.057 | - |
|  | alphaglucanases | 0.397903 | 1 | - |
|  | cello/xylobiases | 0.397903 | 0.965 | - |
|  | **xylanases/xyloglucanases** | **0.0169385** | **0** | 3.39 |
|  | **mannanases** | **0.0409579** | **0** | 8.19 |
|  | **arabinogalactanases** | **0.0298494** | **0** | 5.97 |
|  | **betaglucanases** | **0.0547152** | **0.003** | 10.94 |
|  | pectinases | 0.397903 | 0.612 | - |
|  | chitinases | 0.0726239 | 0.061 | - |
|  | **LPMOs** | **0.0203338** | **0.038** | 4.07 |
| Planctomycetota | **cellulases** | **0.0430123** | **0** | 8.6 |
|  | alphaglucanases | 0.273965 | 0.886 | - |
|  | **cello/xylobiases** | **0.0555284** | **0** | 11.11 |
|  | **xylanases/xyloglucanases** | **0.0635845** | **0** | 12.72 |
|  | **mannanases** | **0.02492** | **0** | 4.99 |
|  | **arabinogalactanases** | **0.0270103** | **0.001** | 5.40 |
|  | **betaglucanases** | **0.0442544** | **0** | 8.85 |
|  | **pectinases** | **0.0700236** | **0** | 14.0 |
|  | **chitinases** | **0.0474387** | **0** | 9.49 |
|  | **LPMOs** | **0.0336677** | **0.042** | 6.73 |
| Chloroflexota | **cellulases** | **0.0671794** | **0** | 13.43 |
|  | **alphaglucanases** | **0.0772826** | **0.001** | 15.45 |
|  | **cello/xylobiases** | **0.120667** | **0** | 24.13 |
|  | **xylanases/xyloglucanases** | **0.0297642** | **0.028** | 5.95 |
|  | **mannanases** | **0.069252** | **0** | 13.85 |
|  | **arabinogalactanases** | **0.0695214** | **0.015015** | 13.90 |
|  | **betaglucanases** | **0.0437064** | **0.004** | 8.74 |
|  | **pectinases** | **0.0634621** | **0** | 12.69 |
|  | **chitinases** | **0.0758622** | **0** | 15.17 |
|  | LPMOs | **-** | **-** | - |
| Bacteroidota | **cellulases** | **0.0223403** | **0** | 4.47 |
|  | alphaglucanases | 1.26666 | 0.853 | - |
|  | cello/xylobiases | 1.26666 | 0.998 | - |
|  | xylanases/xyloglucanases | 0.0209786 | 0.054 | - |
|  | mannanases | 0.0125221 | 0.31 | - |
|  | **arabinogalactanases** | **0.0220927** | **0** | 4.42 |
|  | **betaglucanases** | **0.0510456** | **0** | 10.21 |
| **Phyla** | **Trait** | **Mean genetic depth (τ_D_)** | ***P*-value** | **Percentage of divergence in the 16S rRNA gene** |
| Bacteroidota | **pectinases** | **0.032329** | **0** | 6.47 |
|  | **chitinases** | **0.045098** | **0** | 9.02 |
|  | LPMOs | 0.00783292 | 0.352 | - |
| Actinobacteriota | **cellulases** | **0.0115073** | **0** | 2.3 |
|  | **alphaglucanases** | **0.0427993** | **0** | 8.56 |
|  | cello/xylobiases | 0.151101 | 0.428 | - |
|  | xylanases/xyloglucanases | 0.00542736 | 0.133 | - |
|  | **mannanases** | **0.00994575** | **0** | 1.99 |
|  | arabinogalactanases | 0.00593649 | 0.104 | - |
|  | **betaglucanases** | **0.0133357** | **0** | 2.67 |
|  | pectinases | 0.00561899 | 0.1 | - |
|  | **chitinases** | **0.0195871** | **0** | 3.92 |
|  | **LPMOs** | **0.0257105** | **0.014** | 5.14 |
| Acidobacteriota | **cellulases** | **0.0183912** | **0.001** | 3.68 |
|  | alphaglucanases | 0.229811 | 1 | - |
|  | cello/xylobiases | 0.229811 | 0.998 | - |
|  | **xylanases/xyloglucanases** | **0.0282017** | **0** | 5.64 |
|  | **mannanases** | **0.0258856** | **0** | 5.18 |
|  | arabinogalactanases | 0.0166034 | 0.076 | - |
|  | **betaglucanases** | **0.021114** | **0** | 4.22 |
|  | **pectinases** | **0.0288043** | **0** | 5.76 |
|  | **chitinases** | **0.0481841** | **0** | 9.67 |
|  | LPMOs | - | - | - |
| Proteobacteria | **cellulases** | **0.00978721** | **0** | 1.96 |
|  | **alphaglucanases** | **0.0147869** | **0** | 2.96 |
|  | cello/xylobiases | 0.232397 | 1 | - |
|  | xylanases/xyloglucanases | 0.0086128 | 0 | - |
|  | **mannanases** | **0.00489549** | **0.02** | 0.98 |
|  | arabinogalactanases | 0.0042042 | 0.099 | - |
|  | **betaglucanases** | **0.00846417** | **0** | 1.69 |
|  | **pectinases** | **0.00605161** | **0** | 1.21 |
|  | **chitinases** | **0.00506015** | **0** | 1.01 |
|  | LPMOs | 0.00227247 | 0.493 | - |
| Firmicutes_A | **cellulases** | **0.000777729** | **0.021** | 0.15 |
|  | alphaglucanases | 0.00422742 | 0.522 | - |
|  | cello/xylobiases | 0.120959 | 0.767 | - |
|  | xylanases/xyloglucanases | 0.000661888 | 0.118 | - |
|  | **mannanases** | **0.000851689** | **0.032** | 0.17 |
|  | arabinogalactanases | 0.000851689 | 0.511 | - |
|  | **betaglucanases** | **0.000815743** | **0.047** | 0.16 |
|  | **pectinases** | **0.000993432** | **0** | 0.19 |
|  | **chitinases** | **0.00257905** | **0** | 0.51 |
|  | LPMOs | - | - | - |
| **Phyla** | **Trait** | **Mean genetic depth (τ_D_)** | ***P*-value** | **Percentage of divergence in the 16S rRNA gene** |
| Firmicutes | **cellulases** | **0.0217205** | **0.007** | 4.3441 |
|  | alphaglucanases | 0.517185 | 0.994 | - |
|  | **cello/xylobiases** | **0.0733113** | **0** | 14.66226 |
|  | xylanases/xyloglucanases | 0.00660505 | 0.486 | - |
|  | **mannanases** | **0.0412286** | **0.0327198** | 8.24572 |
|  | **arabinogalactanases** | **0.0259314** | **0** | 5.18628 |
|  | **betaglucanases** | **0.0344543** | **0** | 6.89086 |
|  | pectinases | 0.031574 | 0.843 | - |
|  | **chitinases** | **0.031574** | **0** | 6.3148 |
|  | LPMOs | 0.00195659 | 0.945 | - |
| Firmicutes_C | cellulases | 0.00103579 | 0.155 | - |
|  | alphaglucanases | 0.00246469 | 0.199 | - |
|  | **cello/xylobiases** | **0.0336614** | **0** | 6.73228 |
|  | xylanases/xyloglucanases | - | - | - |
|  | **mannanases** | **0.00797769** | **0.0294464** | 1.595538 |
|  | **arabinogalactanases** | **0.0100116** | **0.024** | 2.00232 |
|  | betaglucanases | 0.00235859 | 0.076 | - |
|  | **pectinases** | **0.0807724** | **0** | 16.15448 |
|  | **chitinases** | **0.0493754** | **0** | 9.87508 |
|  | LPMOs | - | - | - |
| Desulfobacterota | cellulases | 0.00380385 | 0.095 | - |
|  | alphaglucanases | 0.0865495 | 0.901 | - |
|  | **cello/xylobiases** | **0.00631013** | **0** | 1.262026 |
|  | xylanases/xyloglucanases | - | - | - |
|  | mannanases | 0.000724847 | 0.612 | - |
|  | arabinogalactanases | - | - | - |
|  | betaglucanases | 0.000844612 | 0.553 | - |
|  | pectinases | - | - | - |
|  | chitinases | 0.00202953 | 0.216516 | - |
|  | LPMOs | - | - | - |
| Desulfobacterota_A | cellulases | - | - | - |
|  | alphaglucanases | 0.136209 | 1 |  |
|  | **cello/xylobiases** | **0.0130368** | **0** | 2.60736 |
|  | xylanases/xyloglucanases | - | - | - |
|  | mannanases | - | - | - |
|  | arabinogalactanases | - | - | - |
|  | **betaglucanases** | **0.00909777** | **0.0127758** | 1.819554 |
|  | pectinases | - | - | - |
|  | chitinases | - | - | - |
|  | LPMOs | - | - | - |
| Armatinomonadota | cellulases | 0.0617453 | 0.505 | - |
|  | alphaglucanases | 0.302684 | 0.981 | - |
|  | cello/xylobiases | 0.302684 | 0.983 | - |
| **Phyla** | **Trait** | **Mean genetic depth (τ_D_)** | ***P*-value** | **Percentage of divergence in the 16S rRNA gene** |
| Armatinomonadota | xylanases/xyloglucanases | 0.0555677 | 0.121 | - |
|  | mannanases | 0.122185 | 0.271 | - |
|  | **arabinogalactanases** | **0.0300377** | **0.023** | 6.00754 |
|  | betaglucanases | 0.302684 | 0.86 | - |
|  | pectinases | 0.302684 | 1 | - |
|  | chitinases | 0.302684 | 0.659 | - |
|  | LPMOs | - | - | - |
| Campylobacterota | cellulases | - | - | - |
|  | **alphaglucanases** | **0.110649** | **0.0140187** | 22.1298 |
|  | cello/xylobiases | 0.0214273 | 0.14 | - |
|  | xylanases/xyloglucanases | - | - | - |
|  | mannanases | - | - | - |
|  | arabinogalactanases | - | - | - |
|  | betaglucanases | 0.00740487 | 0.212722 | - |
|  | pectinases | 0.00138169 | 0.967 | - |
|  | chitinases | 0.00133822 | 0.613613 | - |
|  | LPMOs | - | - | - |
| Cyanobacteria | cellulases | 0.0256947 | 0.462 | - |
|  | alphaglucanases | 0.213229 | 1 | - |
|  | cello/xylobiases | 0.213229 | 1 | - |
|  | **xylanases/xyloglucanases** | **0.117144** | **0.012** | 23.4288 |
|  | mannanases | 0.0180838 | 0.642 | - |
|  | arabinogalactanases | - | - | - |
|  | betaglucanases | 0.0125802 | 0.714142 | - |
|  | pectinases | 0.0303571 | 0.27918 | - |
|  | chitinases | 0.0571575 | 0.091 | - |
|  | LPMOs | - | - | - |
| Deinococcota | cellulases |  | - |  |
|  | alphaglucanases | 0.0166465 | 1 |  |
|  | cello/xylobiases | 0.0166465 | 0.963 |  |
|  | **xylanases/xyloglucanases** | **0.217155** | **0.01849** | 43.431 |
|  | **mannanases** | **0.217155** | **0.0266876** | 43.431 |
|  | **arabinogalactanases** | **0.217155** | **0.0189873** | 43.431 |
|  | **betaglucanases** | **0.217155** | **0.0241158** | 43.431 |
|  | **pectinases** | **0.217155** | **0.0158479** | 43.431 |
|  | **chitinases** | **0.217155** | **0.0145396** | 43.431 |
|  | LPMOs | - | - | - |
| Marinisomatota | **cellulases** | **0.0597464** | **0** | 11.94928 |
|  | **alphaglucanases** | **0.0485971** | **0.009** | 9.71942 |
|  | cello/xylobiases | 0.171366 | 1 | - |
|  | **xylanases/xyloglucanases** | **0.0703354** | **0.00685714** | 14.06708 |
|  | **mannanases** | **0.0273468** | **0.043** | 5.46936 |
|  | arabinogalactanases | 0.0210604 | 0.157225 | - |
| **Phyla** | **Trait** | **Mean genetic depth (τ_D_)** | ***P*-value** | **Percentage of divergence in the 16S rRNA gene** |
| Marinisomatota | **betaglucanases** | **0.0628336** | **0** | 12.56672 |
|  | **pectinases** | **0.038886** | **0.0285132** | 7.7772 |
|  | chitinases | 0.171366 | 0.95 | - |
|  | LPMOs | - | - | - |
| Myxococcota | **cellulases** | **0.0789279** | **0** | 15.78558 |
|  | alphaglucanases | 0.164795 | 0.604 | - |
|  | cello/xylobiases | 0.164795 | 1 | - |
|  | **xylanases/xyloglucanases** | **0.0468632** | **0.00603015** | 9.37264 |
|  | **mannanases** | **0.0436951** | **0** | 8.73902 |
|  | **arabinogalactanases** | **0.0468632** | **0.0040282** | 9.37264 |
|  | **betaglucanases** | **0.0362074** | **0.001** | 7.24148 |
|  | **pectinases** | **0.0437867** | **0.001** | 8.75734 |
|  | **chitinases** | **0.0317483** | **0.022** | 6.34966 |
|  | LPMOs | - | - | - |
| Nitrospirota | cellulases |  | - | - |
|  | alphaglucanases | 0.288374 | 0.917 | - |
|  | cello/xylobiases | 0.0940241 | 0.504 | - |
|  | xylanases/xyloglucanases | - | - | - |
|  | mannanases | - | - | - |
|  | arabinogalactanases | - | - | - |
|  | betaglucanases | - | - | - |
|  | pectinases | 0.288374 | 1 | - |
|  | chitinases | 0.0430111 | 0.211968 | - |
|  | LPMOs | - | - | - |
| Spirochaetota | **cellulases** | **0.0657621** | **0.024** | 13.15242 |
|  | alphaglucanases | 0.298553 | 1 | - |
|  | cello/xylobiases | 0.298553 | 0.999 | - |
|  | xylanases/xyloglucanases | 0.050113 | 0.109879 | - |
|  | mannanases | 0.0512333 | 0.126012 | - |
|  | arabinogalactanases | 0.0506457 | 0.108333 | - |
|  | **betaglucanases** | **0.061655** | **0.037** | 12.331 |
|  | **pectinases** | **0.0619238** | **0** | 12.38476 |
|  | **chitinases** | **0.0929264** | **0.007** | 18.58528 |
|  | LPMOs | - | - | - |
| Thermotogota | **cellulases** | **0.00724766** | **0.02** | 1.449532 |
|  | alphaglucanases | 0.151661 | 1 | - |
|  | cello/xylobiases | 0.151661 | 1 | - |
|  | xylanases/xyloglucanases | 0.00646088 | 0.073 | - |
|  | mannanases | 0.00552693 | 0.083 | - |
|  | **arabinogalactanases** | **0.00881392** | **0.004** | 1.762784 |
|  | **betaglucanases** | **0.0113095** | **0.021** | 2.2619 |
|  | **pectinases** | **0.0428499** | **0.027** | 8.56998 |
|  | chitinases | 0.00587453 | 0.059 | - |
| **Phyla** | **Trait** | **Mean genetic depth (τ_D_)** | ***P*-value** | **Percentage of divergence in the 16S rRNA gene** |
| Thermotogota | LPMOs | - | - | - |
