# Supplementary material for: Global Distribution of Carbohydrate Utilization Potential in the Prokaryotic Tree of Life
Source: mSystems. 2022 Nov 22;7(6):e00829-22. doi: 10.1128/msystems.00829-22 (PMC9765126; doi:10.1128/msystems.00829-22)
Supplement: TABLE S3 [file msystems.00829-22-s0008.docx]

Supplementary Table S3. The CAZyme families/subfamilies were grouped according the main enzymatic activities characterized in the family/subfamily into 14 different classes or general activities.

| General Activity | CAZyme families | Main activity in the family/subfamily |
| --- | --- | --- |
| **Cellulases** | GH5_1 | endoglucanase (EC 3.2.1.4) |
|  | GH5_2 | endoglucanase (EC 3.2.1.4) |
|  | GH5_4 | endoglucanase (EC 3.2.1.4) |
|  | GH5_5 | endoglucanase (EC 3.2.1.4) |
|  | GH5_25 | endoglucanase (EC 3.2.1.4) |
|  | GH5_26 | endoglucanase (EC 3.2.1.4) |
|  | GH5_37 | endoglucanase (EC 3.2.1.4) |
|  | GH5_38 | endoglucanase (EC 3.2.1.4) |
|  | GH5_39 | endoglucanase (EC 3.2.1.4) |
|  | GH5_46 | endoglucanase (EC 3.2.1.4) |
|  | GH5_52 | exo-β-1,4-glucanase / cellodextrinase (EC 3.2.1.74) |
|  | GH5_53 | exo-β-1,4-glucanase / cellodextrinase (EC 3.2.1.74) |
|  | GH6 | cellobiohydrolase (EC 3.2.1.91) |
|  | GH7 | reducing end-acting cellobiohydrolase (EC 3.2.1.176) |
|  | GH8 | endoglucanase (EC 3.2.1.4); endo-β-1,4-xylanase (EC 3.2.1.8) |
|  | GH9 | endoglucanase (EC 3.2.1.4) |
|  | GH12 | endoglucanase (EC 3.2.1.4) |
|  | GH44 | endoglucanase (EC 3.2.1.4); endo-β-1,4-mannanase (EC 3.2.1.78) |
|  | GH45 | endoglucanase (EC 3.2.1.4) |
|  | GH48 | reducing end-acting cellobiohydrolase (EC 3.2.1.176); endoglucanase (EC 3.2.1.4) |
|  | GH124 | endoglucanase (EC 3.2.1.4) |
| **Chitinases** | GH5_48 | N-acetyl β-glucosaminidase (EC 3.2.1.52); chitosanase (EC 3.2.1.132) |
|  | GH18 | chitinase (EC 3.2.1.14) |
|  | GH19 | chitinase (EC 3.2.1.14) |
|  | GH20 | N-acetyl β-glucosaminidase (EC 3.2.1.52) |
|  | GH46 | chitosanase (EC 3.2.1.132) |
|  | GH75 | chitosanase (EC 3.2.1.132) |
| **Alpha-glucanases** | GH13 | α-amylase (EC 3.2.1.1) |
|  | GH14 | β-amylase (EC 3.2.1.2) |
|  | GH15 | glucoamylase (EC 3.2.1.3) |
|  | GH31 | α-glucosidase (EC 3.2.1.20) |
|  | GH57 | α-amylase (EC 3.2.1.1); amylopullulanase (EC 3.2.1.41) |
|  | GH66 | dextranase (EC 3.2.1.11) |
|  | GH68 | levansucrase (EC 2.4.1.10); β-fructofuranosidase (EC 3.2.1.26) |
|  | GH70 | dextransucrase (EC 2.4.1.5) |
| General Activity | CAZyme families | Main activity in the family/subfamily |
| Alpha-glucanases | GH77 | amylomaltase or 4-α-glucanotransferase (EC 2.4.1.25) |
|  | GH100 | β-fructofuranosidase (EC 3.2.1.26) |
|  | GH119 | α-amylase (EC 3.2.1.1) |
|  | GH122 | α-glucosidase (EC 3.2.1.20) |
|  | GH126 | α-amylase (EC 3.2.1.1) |
|  | GH133 | amylo-α-1,6-glucosidase (EC 3.2.1.33) |
| **Beta-glucosidases/**  **beta-xylosidases** | GH1 | β-glucosidase (EC 3.2.1.21) |
|  | GH3 | β-glucosidase (EC 3.2.1.21) |
|  | GH5_22 |  |
|  | GH5_43 | β-glucosidase (EC 3.2.1.21) |
|  | GH5_45 | β-glucosidase (EC 3.2.1.21) |
|  | GH30_2 | β-xylosidase (EC 3.2.1.37) |
|  | GH30_6 | β-glucosidase (EC 3.2.1.21) |
|  | GH39 | β-xylosidase (EC 3.2.1.37) |
|  | GH43_1 | β-xylosidase (EC 3.2.1.37) |
|  | GH43_11 | β-xylosidase (EC 3.2.1.37) |
|  | GH43_22 | β-xylosidase (EC 3.2.1.37) |
|  | GH43_27 | β-xylosidase (EC 3.2.1.37) |
|  | GH43_35 | β-xylosidase (EC 3.2.1.37) |
|  | GH52 | β-xylosidase (EC 3.2.1.37) |
|  | GH116 | β-glucosidase (EC 3.2.1.21); β-xylosidase (EC 3.2.1.37) |
| **Xylanases/xyloglucanases** | GH5_21 | endo-β-1,4-xylanase (EC 3.2.1.8) |
|  | GH5_35 | endo-β-1,4-xylanase (EC 3.2.1.8) |
|  | GH10 | endo-β-1,4-xylanase (EC 3.2.1.8) |
|  | GH11 | endo-β-1,4-xylanase (EC 3.2.1.8) |
|  | GH30_7 | endo-β-1,4-xylanase (EC 3.2.1.8) |
|  | GH30_8 | endo-β-1,4-xylanase (EC 3.2.1.8) |
|  | GH43_16 | endo-β-1,4-xylanase (EC 3.2.1.8) |
|  | GH43_29 | endo-β-1,4-xylanase (EC 3.2.1.8) |
|  | GH67 | xylan α-1,2-glucuronidase (EC 3.2.1.131) |
|  | GH74 | xyloglucanase (EC 3.2.1.151) |
|  | GH115 | xylan α-1,2-glucuronidase (EC 3.2.1.131) |
|  | GH141 | endo-β-1,4-xylanase (EC 3.2.1.8) |
| **Mannanases** | GH5_7 | endo-β-1,4-mannanase (EC 3.2.1.78) |
|  | GH5_8 | endo-β-1,4-mannanase (EC 3.2.1.78) |
|  | GH5_10 | endo-β-1,4-mannanase (EC 3.2.1.78) |
|  | GH5_17 | endo-β-1,4-mannanase (EC 3.2.1.78) |
|  | GH5_18 | β-mannosidase (EC 3.2.1.25) |
|  | GH5_19 | β-mannosidase (EC 3.2.1.25) |
|  | GH5_36 | endo-β-1,4-mannanase (EC 3.2.1.78) |
|  | GH5_40 | endo-β-1,4-mannanase (EC 3.2.1.78) |
|  | GH5_41 | endo-β-1,4-mannanase (EC 3.2.1.78) |
| General Activity | CAZyme families | Main activity in the family/subfamily |
| Mannanases | GH5_55 | endo-β-1,4-mannanase (EC 3.2.1.78) |
|  | GH26 | endo-β-1,4-mannanase (EC 3.2.1.78) |
|  | GH134 | endo-β-1,4-mannanase (EC 3.2.1.78) |
|  | GH164 | β-mannosidase (EC 3.2.1.25) |
| **Arabinogalactanases** | GH5_16 | endo-β-1,6-galactanase (EC:3.2.1.164) |
|  | GH16_10 | endo-β-1,3-galactanase (EC 3.2.1.181) |
|  | GH30_5 | endo-β-1,6-galactanase (EC:3.2.1.164) |
|  | GH43_4 | endo-α-1,5-L-arabinanase (EC 3.2.1.99) |
|  | GH43_5 | endo-α-1,5-L-arabinanase (EC 3.2.1.99) |
|  | GH43_24 | exo-β-1,3-galactanase (EC 3.2.1.145) |
|  | GH43_37 | endo-α-1,5-L-arabinanase (EC 3.2.1.99) |
|  | GH53 | endo-β-1,4-galactanase (EC 3.2.1.89) |
| **Beta-glucanases** | GH5_47 | endo-1,3-β-glucanase (EC 3.2.1.39); endo-1,6-β-glucosidase (EC 3.2.1.75) |
|  | GH16 | endo-1,6-β-glucosidase (EC 3.2.1.75); endo-1,3-β-glucanase (EC 3.2.1.39); lichenase / endo-β-1,3-1,4-glucanase (EC 3.2.1.73) |
|  | GH17 | endo-1,3-β-glucanase (EC 3.2.1.39) |
|  | GH30_3 | endo-1,6-β-glucosidase (EC 3.2.1.75) |
|  | GH55 | exo-β-1,3-glucanase (EC 3.2.1.58); endo-1,3-β-glucanase (EC 3.2.1.39) |
|  | GH64 | endo-1,3-β-glucanase (EC 3.2.1.39) |
|  | GH72 | β-1,3-glucanosyltransglycosylase (EC 2.4.1.-) |
|  | GH81 | endo-1,3-β-glucanase (EC 3.2.1.39) |
|  | GH128 | endo-1,3-β-glucanase (EC 3.2.1.39) |
|  | GH144 | endo-β-1,2-glucanase (EC 3.2.1.71) |
|  | GH148 | β-1,3-glucanase (EC 3.2.1.-) |
|  | GH149 | β-1,3-glucan phosphorylase (EC 2.4.1.97) |
|  | GH152 | endo-1,3-β-glucanase (EC 3.2.1.39) |
|  | GH157 | endo-1,3-β-glucanase (EC 3.2.1.39) |
|  | GH158 | endo-1,3-β-glucanase (EC 3.2.1.39) |
|  | GH161 | β-1,3-glucan phosphorylase (EC 2.4.1.97) |
|  | GH162 | endo-β-1,2-glucanase (EC 3.2.1.71) |
| **Pectinases** | CE8 | pectin methylesterase (EC 3.1.1.11) |
|  | GH105 | unsaturated rhamnogalacturonyl hydrolase (EC 3.2.1.172) |
|  | GH106 | α-L-rhamnosidase (EC 3.2.1.40) |
|  | GH127 | β-L-arabinofuranosidase (EC 3.2.1.185) |
|  | GH137 | β-L-arabinofuranosidase (EC 3.2.1.185) |
|  | GH138 | rhamnogalacturonan α-1,2-galacturonohydrolase (EC 3.2.1.173) |
|  | GH139 | α-2-O-Me-L-fucosidase (EC 3.2.1.-) |
|  | GH140 | β-1,2-apiosidase (EC 3.2.1.-) |
|  | GH142 | β-L-arabinofuranosidase (EC 3.2.1.185) |
|  | GH143 | 2-keto-3-deoxy-D-lyxo-heptulosaric acid hydrolase (EC 3.2.1.-) |
|  |  |  |
| General Activity | CAZyme families | Main activity in the family/subfamily |
| Pectinases | GH145 | L-Rhα-α-1,4-GlcA α-L-rhamnohydrolase (EC 3.2.1.-) |
|  | GH146 | β-L-arabinofuranosidase (EC 3.2.1.185) |
|  | GH151 | α-L-fucosidase (EC 3.2.1.51) |
|  | PL1_1 | pectate lyase (EC 4.2.2.2) |
|  | PL1_2 | pectate lyase (EC 4.2.2.2) |
|  | PL1_3 | pectate lyase (EC 4.2.2.2) |
|  | PL1_5 | pectate lyase (EC 4.2.2.2); exo-pectate lyase (EC 4.2.2.9) |
|  | PL1_6 | pectate lyase (EC 4.2.2.2) |
|  | PL1_7 | pectate lyase (EC 4.2.2.2) |
|  | PL1_8 | pectin lyase (EC 4.2.2.10) |
|  | PL10 | pectate lyase (EC 4.2.2.2) |
|  | PL10_1 | pectate lyase (EC 4.2.2.2) |
|  | PL10_2 | pectate lyase (EC 4.2.2.2) |
|  | PL10_3 | pectate lyase (EC 4.2.2.2) |
|  | PL11 | rhamnogalacturonan endolyase (EC 4.2.2.23); rhamnogalacturonan exolyase (EC 4.2.2.24) |
|  | PL11_1 | rhamnogalacturonan endolyase (EC 4.2.2.23); rhamnogalacturonan exolyase (EC 4.2.2.24) |
|  | PL11_2 | rhamnogalacturonan endolyase (EC 4.2.2.23); rhamnogalacturonan exolyase (EC 4.2.2.24) |
|  | PL26 | rhamnogalacturonan exolyase (EC 4.2.2.24) |
| **Other hemicellulases** | GH2 | β-galactosidase (EC 3.2.1.23) |
|  | GH27 | α-galactosidase (EC 3.2.1.22) |
|  | GH35 | β-galactosidase (EC 3.2.1.23) |
|  | GH36 | α-galactosidase (EC 3.2.1.22) |
|  | GH43_2 | α-L-arabinofuranosidase (EC 3.2.1.55) |
|  | GH43_9 | α-L-arabinofuranosidase (EC 3.2.1.55) |
|  | GH43_10 | α-L-arabinofuranosidase (EC 3.2.1.55) |
|  | GH43_12 | α-L-arabinofuranosidase (EC 3.2.1.55) |
|  | GH43_17 | α-L-arabinofuranosidase (EC 3.2.1.55) |
|  | GH43_26 | α-L-arabinofuranosidase (EC 3.2.1.55) |
|  | GH43_34 | α-L-arabinofuranosidase (EC 3.2.1.55) |
|  | GH51 | α-L-arabinofuranosidase (EC 3.2.1.55) |
|  | GH54 | α-L-arabinofuranosidase (EC 3.2.1.55) |
|  | GH62 | α-L-arabinofuranosidase (EC 3.2.1.55) |
|  | GH97 | α-galactosidase (EC 3.2.1.22) |
|  | GH110 | α-galactosidase (EC 3.2.1.22) |
|  | GH147 | β-galactosidase (EC 3.2.1.23) |
|  | GH165 | β-galactosidase (EC 3.2.1.23) |
|  | CE1 | acetyl xylan esterase (EC 3.1.1.72) |
|  | CE2 | acetyl xylan esterase (EC 3.1.1.72) |
|  | CE3 | acetyl xylan esterase (EC 3.1.1.72) |
| General Activity | CAZyme families | Main activity in the family/subfamily |
| Other hemicellulases | CE4 | acetyl xylan esterase (EC 3.1.1.72) |
|  | CE5 | acetyl xylan esterase (EC 3.1.1.72) |
|  | CE6 | acetyl xylan esterase (EC 3.1.1.72) |
|  | CE7 | acetyl xylan esterase (EC 3.1.1.72) |
|  | CE12 | acetyl xylan esterase (EC 3.1.1.72) |
|  | CE15 | acetyl xylan esterase (EC 3.1.1.72) |
| **Carragenases/agarases** | GH16_11 | β-porphyranase (EC 3.2.1.178) |
|  | GH16_12 | β-porphyranase (EC 3.2.1.178) |
|  | GH16_13 | β-carrageenase (EC 3.2.1.-) |
|  | GH16_14 | β-agarase (EC 3.2.1.81) |
|  | GH16_15 | β-agarase (EC 3.2.1.81) |
|  | GH16_16 | β-agarase (EC 3.2.1.81) |
|  | GH16_17 | κ-carrageenase (EC 3.2.1.83) |
|  | GH16_26 | β-porphyranase (EC 3.2.1.178) |
|  | GH50 | β-agarase (EC 3.2.1.81) |
|  | GH82 | Ι-carrageenase (EC 3.2.1.157) |
|  | GH86 | β-agarase (EC 3.2.1.81) |
|  | GH96 | α-agarase (EC 3.2.1.158) |
|  | GH107 | sulfated fucan endo-1,4-fucanase (EC 3.2.1.-) |
|  | GH117 | α-1,3-L-neoagarooligosaccharide hydrolase (EC 3.2.1.-) |
|  | GH118 | β-agarase (EC 3.2.1.81) |
|  | GH150 | λ-carrageenase (EC 3.2.1.162) |
|  | GH167 | β-carrageenase (EC 3.2.1.-) |
| **Peptidoglycanases** | GH22 | lysozyme (EC 3.2.1.17) |
|  | GH23 | lysozyme (EC 3.2.1.17); peptidoglycan lytic transglycosylase (EC 4.2.2.n1) |
|  | GH24 | lysozyme (EC 3.2.1.17) |
|  | GH25 | lysozyme (EC 3.2.1.17) |
|  | GH73 | lysozyme (EC 3.2.1.17) |
|  | GH102 | peptidoglycan lytic transglycosylase (EC 4.2.2.n1) |
|  | GH103 | peptidoglycan lytic transglycosylase (EC 4.2.2.n1) |
|  | GH104 | peptidoglycan lytic transglycosylase (EC 4.2.2.n1) |
|  | GH108 | lysozyme (EC 3.2.1.17) |
|  | GH153 | Poly-beta-1,6-N-acetyl-D-glucosamine N-deacetylase |
|  | CE9 | N-acetylglucosamine 6-phosphate deacetylase (EC 3.5.1.25); N-acetylglucosamine 6-phosphate deacetylase (EC 3.5.1.80) |
| **Glycoconjugate-degrading enzymes*** | GH5_12 | β-glucosylceramidase (EC 3.2.1.45) |
|  | GH5_27 | endoglycoceramidase (EC 3.2.1.123) |
|  | GH5_28 | endoglycoceramidase (EC 3.2.1.123) |
| General Activity | CAZyme families | Main activity in the family/subfamily |
| Glycoconjugate-degrading enzymes* | GH5_29 | galactosylceramidase (EC 3.2.1.46) |
|  | GH30_4 | β-fucosidase (EC 3.2.1.38) |
|  | GH30_9 | β-glucuronidase (3.2.1.31) |
|  | GH33 | sialidase or neuraminidase (EC 3.2.1.18) |
|  | GH34 | sialidase or neuraminidase (EC 3.2.1.18) |
|  | GH38 | α-mannosidase (EC 3.2.1.24) |
|  | GH43_32 | β-D-galactofuranosidase (EC 3.2.1.146) |
|  | GH43_30 | β-D-galactofuranosidase (EC 3.2.1.146) |
|  | GH47 | mannosyl-oligosaccharide α-1,2-mannosidase (EC 3.2.1.113) |
|  | GH56 | hyaluronidase (EC 3.2.1.35) |
|  | GH58 | endo-N-acetylneuraminidase or endo-sialidase (EC 3.2.1.129) |
|  | GH59 | galactocerebrosidase (EC 3.2.1.46) |
|  | GH76 | α-1,6-mannanase (EC 3.2.1.101) |
|  | GH79 |  |
|  | GH83 | sialidase or neuraminidase (EC 3.2.1.18) |
|  | GH84 | hyaluronidase (EC 3.2.1.35); [protein]-3-O-(GlcNAc)-L-Ser/Thr β-N-acetylglucosaminidase (EC 3.2.1.169) |
|  | GH85 | endo-β-N-acetylglucosaminidase (EC 3.2.1.96) |
|  | GH88 | d-4,5-unsaturated β-glucuronyl hydrolase (EC 3.2.1.-) |
|  | GH89 | α-N-acetylglucosaminidase (EC 3.2.1.50) |
|  | GH92 | α-mannosidase (EC 3.2.1.24) |
|  | GH98 | blood-group endo-β-1,4-galactosidase (EC 3.2.1.102); blood group A- and B-cleaving endo-β-1,4-galactosidase (EC 3.2.1.-) |
|  | GH99 | glycoprotein endo-α-1,2-mannosidase (EC 3.2.1.130) |
|  | GH101 | endo-α-N-acetylgalactosaminidase (EC 3.2.1.97) |
|  | GH109 | α-N-acetylgalactosaminidase (EC 3.2.1.49) |
|  | GH111 | keratan sulfate hydrolase (endo-β-N-acetylglucosaminidase) (EC 3.2.1.-) |
|  | GH114 | endo-α-1,4-polygalactosaminidase (EC 3.2.1.109) |
|  | GH121 | β-L-arabinobiosidase (EC 3.2.1.-) |
|  | GH123 | β-N-acetylgalactosaminidase (EC 3.2.1.53); glycosphingolipid β-N-acetylgalactosaminidase (EC 3.2.1.-) |
|  | GH129 | α-N-acetylgalactosaminidase (EC 3.2.1.49) |
|  | GH135 | α-1,4-galactosaminogalactan hydrolase (EC 3.2.1.-) |
| General Activity | CAZyme families | Main activity in the family/subfamily |
| Glycoconjugate-degrading enzymes* | GH136 | lacto-N-biosidase (EC 3.2.1.140) |
|  | GH154 | β-glucuronidase (3.2.1.31) |
|  | GH156 | sialidase or neuraminidase (EC 3.2.1.18) |
|  | GH159 | β-D-galactofuranosidase (EC 3.2.1.146) |
|  | GH160 | blood-group endo-β-1,4-galactosidase (EC 3.2.1.102); blood group A- and B-cleaving endo-β-1,4-galactosidase (EC 3.2.1.-) |
|  | GH163 | endo-β-N-acetylglucosaminidase cleaving GlcNAc-β-1,2-Man (EC 3.2.1.-) |
|  | GH166 | α-1,4-galactosaminogalactan hydrolase (EC 3.2.1.-) |
| **LPMOs** | AA10 | lytic cellulose monooxygenase (C1-hydroxylating) (EC 1.14.99.54); lytic cellulose monooxygenase (C4-dehydrogenating)(EC 1.14.99.56); lytic chitin monooxygenase (EC 1.14.99.53) |

*Glycoconjugate-degrading enzymes (enzymes acting on glycoproteins, glycolipids, or proteoglycans)
